# Metabolomics Test Materials for Quality Control: A Study of a Urine Materials Suite

**DOI:** 10.3390/metabo9110270

**Published:** 2019-11-07

**Authors:** Daniel W. Bearden, David A. Sheen, Yamil Simón-Manso, Bruce A. Benner, Werickson F. C. Rocha, Niksa Blonder, Katrice A. Lippa, Richard D. Beger, Laura K. Schnackenberg, Jinchun Sun, Khyati Y. Mehta, Amrita K. Cheema, Haiwei Gu, Ramesh Marupaka, G. A. Nagana Gowda, Daniel Raftery

**Affiliations:** 1 Chemical Sciences Division, National Institute of Standards and Technology, Gaithersburg, MD 20899, USA; danbearden@metabolomicspartners.com (D.W.B.); wfrocha@inmetro.gov.br (W.F.C.R.); niksa.blonder@nist.gov (N.B.); katrice.lippa@nist.gov (K.A.L.); 2 Biomolecular Measurement Division, National Institute of Standards and Technology, Gaithersburg, MD 20899, USA; yamil.simon@nist.gov; 3 National Institute of Metrology, Quality, and Technology—INMETRO, 25250-020 Duque de Caxias, RJ, Brazil; 4 Division of Systems Biology, National Center for Toxicological Research, U.S. Food and Drug Administration, Jefferson, AR 72079, USA; richard.beger@fda.hhs.gov (R.D.B.); laura.schnackenberg@fda.hhs.gov (L.K.S.); jinchun.sun@fda.hhs.gov (J.S.); 5 Department of Oncology, Lombardi Comprehensive Cancer Center, Georgetown University Medical Center, Washington, DC 20057, USA; kym8@georgetown.edu (K.Y.M.); akc27@georgetown.edu (A.K.C.); 6 Departments of Oncology and Biochemistry, Molecular and Cellular Biology, Georgetown University Medical Center, Washington, DC 20057, USA; 7 College of Health Solutions, Arizona State University, Phoenix, AZ 85004, USA; haiweigu@asu.edu; 8 Clinical Toxicology at CIAN Diagnostics, Frederick, MD 21703, USA; rameshmrpk@gmail.com; 9 Department of Anesthesiology and Pain Medicine, Mitochondria and Metabolism Center, University of Washington, Seattle, WA 98109, USA; ngowda@uw.edu (G.A.N.G.); draftery@uw.edu (D.R.)

**Keywords:** interlaboratory study, nuclear magnetic resonance, chromatography, principal components analysis, reproducibility

## Abstract

There is a lack of experimental reference materials and standards for metabolomics measurements, such as urine, plasma, and other human fluid samples. Reasons include difficulties with supply, distribution, and dissemination of information about the materials. Additionally, there is a long lead time because reference materials need their compositions to be fully characterized with uncertainty, a labor-intensive process for material containing thousands of relevant compounds. Furthermore, data analysis can be hampered by different methods using different software by different vendors. In this work, we propose an alternative implementation of reference materials. Instead of characterizing biological materials based on their composition, we propose using untargeted metabolomic data such as nuclear magnetic resonance (NMR) or gas and liquid chromatography-mass spectrometry (GC-MS and LC-MS) profiles. The profiles are then distributed with the material accompanying the certificate, so that researchers can compare their own metabolomic measurements with the reference profiles. To demonstrate this approach, we conducted an interlaboratory study (ILS) in which seven National Institute of Standards and Technology (NIST) urine Standard Reference Material^®^s (SRM^®^s) were distributed to participants, who then returned the metabolomic data to us. We then implemented chemometric methods to analyze the data together to estimate the uncertainties in the current measurement techniques. The participants identified similar patterns in the profiles that distinguished the seven samples. Even when the number of spectral features is substantially different between platforms, a collective analysis still shows significant overlap that allows reliable comparison between participants. Our results show that a urine suite such as that used in this ILS could be employed for testing and harmonization among different platforms. A limited quantity of test materials will be made available for researchers who are willing to repeat the protocols presented here and contribute their data.

## 1. Introduction

A recent survey of practitioners in metabolomics research indicated an acute need for experimental reference materials [1]. The lack of reasonably priced, widely available, complex matrix test materials reduces the opportunity for robust quality control and interlaboratory harmonization. This lack is a real impediment to validation of methods and large-scale application of metabolomics in epidemiologic-scale studies.

While it is conceivable that individuals, private entities, or university laboratories could produce such test materials, there may be significant difficulties with longevity of the supply, communication of relevant information about the materials, and distribution of the materials. The National Institute of Standards and Technology (NIST) is the national metrology institute (NMI) for the USA and has historically filled the role of providing standard reference materials when entities such as trade groups, scientific organizations, or government entities indicate an industry-wide need for such materials (www.nist.gov/srm). To that end, we conducted an interlaboratory study (ILS) intended to address some of the issues related to test materials in the field of metabolomics and lay the groundwork for a stable, affordable, and accessible supply of suitable test materials, information and findings.

This work’s ILS embodies an emerging concept regarding quality control (QC) materials that contrasts with traditional certified reference materials (CRMs; SRMs are CRMs produced by NIST) in a few specific ways that may be beneficial in a rapidly evolving research area like metabolomics. In contrast to CRMs, QC materials would be property-assigned, including compound identification, through consensus of the community. This will be extremely valuable as organizations and regulatory bodies settle on platform-specific protocols that may have different data quality requirements. By allowing practitioners in different application areas to contribute to consensus evaluations and protocols using QC materials as proposed here, consistency and harmonization between laboratories can arise while providing for improved data quality in specific applications. QC materials would, therefore, be suitable for use as reference materials within the scope they were designed for [2].

Another distinction between CRMs and QC materials would be the ‘time to market’ for replacement or alternative materials based on community demand and inventory levels for the materials. A large part of the long development time for CRMs is the requirement for full, multi-platform validation of the materials with certified value assignment before they are released with a certificate of analysis (COA) by their producers [3]. It is much easier in the QC material paradigm to perform some smaller number of qualifying tests for a QC material and release it into the consensus-building community compared to the strict data quality and validation requirements for producing a CRM [4].

This study envisions a quality data repository where data from QC materials analyzed within an ILS can be deposited systematically, compared with consensus results from other laboratories, and integrated into the consensus values that the community develops. The public availability of raw and semi-processed data from a consistent set of samples analyzed in different labs on different instruments may provide a valuable dataset for demonstration of developmental bioinformatics analysis tools for inter- and intra-laboratory data quality metrics.

NIST has identified and procured an initial set of human urine matrix SRMs that could prove useful in urine metabolomics studies as QC and method development materials. The materials include a set of nine frozen and pooled urine samples. More information about the pooled samples can be found in the COAs for the SRMs [5,6,7,8]. This set of samples could provide a basis for demonstrating not only consistent analytical results on individual samples, but also inter-sample consistency for classification and pattern recognition [4]. Utilizing a ‘natural material’ which contains all the potential matrix-related complications in the various metabolomics platforms is essential for producing robust, meaningful methods of measurement and analysis that can propel the field forward. It should be noted, as stated in our ethics statement, that the development of human-tissue SRMs is performed in accordance with the usual standards for sampling human tissue, including standards of informed consent, and the development or use of further QC material suites will need to follow these same standards.

## 2. Materials and Methods 

### 2.1. Interlaboratory Study Design

In this ILS, a small number of NIST and non-NIST experts were identified that have expertise in the analytical platforms most commonly used in metabolomics studies: Nuclear magnetic resonance spectroscopy (NMR), gas chromatography mass spectrometry (GC-MS) and liquid chromatography mass spectrometry (LC-MS). Frozen test materials were shipped overnight, along with corresponding materials such as appropriate calibration solutions and buffers. Detailed, platform-specific protocols were developed for analysis of the samples with standardized reporting forms. Participants were requested to follow the procedures as closely as possible, but a mechanism for discussing and clarifying the protocols was used to address any ambiguities that became apparent. Participants are only identified by a code in all public reporting of the results. Analysis results and raw data were delivered to NIST for analysis as described elsewhere in this manuscript.

It should be mentioned that some degree of coordination with the participants was needed in order to properly convey and apply the experimental protocol (attached in the Supplemental Information). This protocol was designed to ensure the data would meet the minimum reporting standards for metabolomics data [9]. The emphasis of the study we report is on the set of materials; that is, our aim is to demonstrate that these urine matrices show subtle yet measurable differences which can be detected by a variety of instruments and allow us to separate and group them using statistical techniques. This study is more limited than traditional metabolomics work; among other limitations, there is no biological sampling because we are using pre-made reference materials, and there is only a limited set of techniques used (e.g., only reversed-phase chromatography or 1-D proton NMR). 

As part of the collection process for NIST human-tissue Standard Reference Materials, all donors gave their informed consent. The study was conducted in accordance with United States regulations 15 CFR 27 and 45 CFR 46 and the protocol was approved by the NIST Human Subjects Protection Office with project code MML-16-0022.

### 2.2. Sample Descriptions

In the development of human tissue or fluid SRMs, NIST identifies a desired population and an outside organization is tasked with screening donors, collecting samples, and pooling the samples. The pooled material is thus an average across the population it is supposed to represent. NIST conducts or supervises any analysis that is to be performed.

Seven distinct materials were distributed in the ILS. One of the materials was replicated three times for a total of nine samples. The samples were human urine pools from four SRMs and one research-grade test material (RGTM):

- SRM 3667 [5] is a single pool of human urine collected from healthy male and female donors;
- SRM 3672 [6] is single pool of human urine collected from donors who smoked more than one pack of cigarettes a day;
- SRM 3673 [7] is single pool of human urine collected from non-smoker donors who were not exposed to secondhand cigarette smoke;
- SRM 3671 [8] is composed of three different pools, each collected from donors from a different exposure population: smokers who smoked at least one pack of cigarettes a day, nonsmokers exposed to secondhand cigarette smoke, and nonsmokers not exposed to cigarette smoke;
- The RGTM is a version of the SRM 3673 material with an additive. The additive does not produce a response in most analytical platforms and so this material serves as a functional replicate of SRM 3673.

To preserve the blinded nature of the ILS, we labelled the samples A through I, and will henceforth refer to them by these codes.

### 2.3. Sample Preparation Protocols

#### 2.3.1. NMR Protocol Summarized

The experimental protocol for the NMR platform included basic and optional exercises. Three participants completed the basic exercises and reporting. Additionally, some data for the optional exercises was delivered. The three laboratories each measured spectra at different field strengths, one laboratory at 600 MHz, one at 700 MHz, and the third at 800 MHz. The basic analysis included sample preparation with a provided buffer solution (volumetric dilution with mass determinations), 1D ^1^H analysis of the nine urine samples, instrument temperature calibration, and an NMR pulse experiment (1D NOSEY sequence with presaturation of the residual water signal).

In addition to spectra of the nine samples included in the ILS, participants were directed to acquire seven additional spectra, a blank (i.e., buffer only) and six additional replicates of Sample A, for a total of 16 NMR basic spectra. Participants used data processing tools of their choice if the identified analysis constraints could be applied. The basic data analysis included a standard signal-to-noise calculation for each of the spectra. The optional components of the NMR ILS included collection of 2D JRES spectra, corresponding analyses and an optional ‘compound ID’ challenge on one of the samples.

The amount of effort requested in the ILS was too extensive, based on the limited response to the optional exercises and the time constraints requested in the ILS. There was not enough participation among the labs to do any additional assessment of the optional parts of the protocol.

#### 2.3.2. LC-MS Protocol Summarized

Like the NMR platform, the LC-MS experimental protocol included basic and optional exercises. Five participants completed the basic exercises. The instrumentation used covers a variety of LC/MS systems, including Waters Acquity UPLC coupled to Waters Xevo TQ-S (Waters Corporation, Milford, MA, USA), Thermo Dionex U3000 coupled to an Orbitrap Fusion-Lumos (Thermo Fisher Scientific, Waltham, MA, USA), Agilent HPLC 1290 coupled to either a Thermo QExactive or an Agilent QToF 6530 (Agilent Technologies, Santa Clara, CA, USA), making this inter-laboratory comparison representative of current LC-MS technologies in the metabolomics area. The LC-MS protocol was adapted from previous studies [10,11,12] with minor modifications for optimizing performance on a variety of LC-MS platforms.

Briefly, the sample preparation procedure consisted of four simple steps (i) urine samples were withdrawn from −80 °C and left at room temperature to thaw, (ii) the samples were then treated with methanol (1:1, *v*/*v*) and centrifuged, (iii) the supernatant was collected and subjected to evaporation in a nitrogen current to remove methanol, and (iv) reconstituted with 200 µL of 1% acetonitrile and 0.1% formic acid previously spiked with a labeled compound. The LC-MS analysis was performed using reversed-phase chromatography. The experiments were blinded and randomized, and replicates were included. Certain constraints were applied to the basic LC analysis derived from the use of a column provided by the organizers. The gradient table, flow rate, injection volume and temperature were optimized for this column. MS settings constraints were kept at a minimum, so optimized settings for maximum coverage on each instrument were used. For details, see the LC-MS annex of the protocol.

Four participants also completed the optional exercises. 

#### 2.3.3. GC-MS Protocol Summarized

Two participants contributed raw data for gas chromatography/mass spectrometric (GC-MS) measurements of the nine urine samples including tentative identifications from a mass spectral database search. These labs also provided measurements of the Grob test mixture [13] and process/preparation blanks using the method protocols described below. Prospective participants were encouraged to use previously defined sample preparation and analysis procedures [14].

Briefly, 100 µL to 200 µL aliquots of urine were weighed and incubated with 20 µL of a urease suspension for 1 h at 37 °C. Methanol (1.7 mL) was added, mixed, and centrifuged (10 min at 1047 rad/sec (10,000 rpm) at 4 °C) to precipitate any residual urease. The supernatant was transferred to glass tubes, evaporated to dryness, reconstituted with 100 µL toluene (dried over anhydrous sodium sulfate), and again taken to dryness. The sample residues were then treated with 50 µL methoxylamine for a 2 h incubation at 60 °C, followed by rapid addition of 100 µL N-methyl-N-(trimethylsilyl) trifluoroacetamide (MSTFA) with 1% trichloromethylsiloxane (TCMS), for a 1 h incubation at 60 °C. The resulting trimethylsilyl (TMS) derivatizations were allowed to cool and 100 µL aliquots transferred to GC vials.

Lab 1 employed a 60 m × 0.25 mm (ID) ZB-5ms column (0.25 µm phase thickness, Phenomenex, Torrance, CA) operated at a constant flow of 2 mL/min helium (average linear velocity = 37.8 cm/s) for a 0.5 µL on-column injection. The temperature program began at 130 °C (1 min hold) and a 5 °C/min ramp to 300 °C and a 10 min hold at this final temperature (run time = 79.3 min). The MS was scanned from *m*/*z* 30–600 with a source, quadrupole, and interface temperature of 200 °C, 150 °C, and 320 °C, respectively. Each of the triplicate preparations of the nine samples was injected once, along with multiple injections of preparation blanks and the Grob test mixture throughout the GC-MS sequence. Mass spectra of chromatographic peaks not attributable to the process/preparation blanks were searched against the NIST Mass Spectral Database (ver. 2.2, June 10, 2014). This database contains many mass spectra for TMS derivatives of polar organic compounds including sugars and other carbohydrates.

Lab 2 precipitated protein in the urine with methanol and derivatized as described above. For measurement of the untargeted species, the external participant used an Agilent DB5-MS+10m Duraguard Capillary Column (30 m × 250 μm × 0.25 μm). The column temperature was maintained at 60 °C for 1.00 min, then increased at a rate of 10 °C/min to 325 °C and held at this temperature for 10 min. Mass spectral signals were recorded after a 4.90 min solvent delay.

### 2.4. Chemometric Analysis

**Figure 1:**
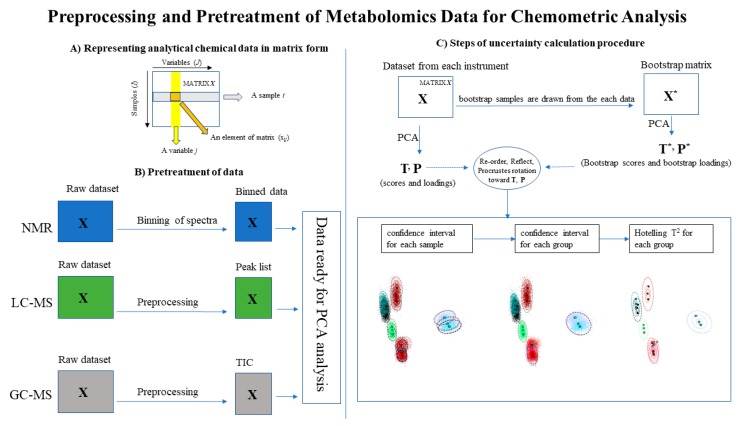
Preprocessing and pretreatment of the experimental data. (**A**) The matrix representation of the analytical chemical data. (**B**) The pretreatment of the raw data resulting in the processed data used for analysis. In the case of liquid chromatography mass spectrometry (LC-MS), the preprocessed data is a list of identified features. For nuclear magnetic resonance spectroscopy (NMR), the preprocessed data is binned spectra. For gas chromatography mass spectrometry (GC-MS), the preprocessed data is a total ion chromatogram (TIC). (**C**) A schematic of the uncertainty calculation using bootstrapping, described in Appendix A. The data matrix **X** is analyzed using principal components analysis (PCA), generating scores **T** and loadings **P**. Bootstrap matrices X* are sampled from **X**, generating bootstrap scores T* and loadings P*. The scores are aligned using an orthogonal Procrustes algorithm to estimate uncertainty in classifications.

The output of the measurements from the various platforms were analyzed for consistency using principal components analysis (PCA) with uncertainty analysis. In keeping with the goal of QC materials characterized by analytical profiles rather than by a defined composition, we have not attempted to assign an identity to any of the features in the profiles and PCA is done only on the intensity data. Ideally, we would perform consistency analysis as done in previous studies [4], but there is not enough data from the ILS for this analysis. However, for the LC-MS analysis, putative assignments were given for overlapping features that were found in all four labs. Our analysis is summarized below, presented graphically in Figure 1, and is described in more detail in the mathematical Appendix A. All analysis was performed in Python and computer codes used in this work are included in Supplemental Information, as is the anonymized data. Software libraries used include scikit-learn [15] and the machine learning uncertainty toolkit (pages.nist.gov/mL_uncertainty_py).

#### 2.4.1. Preprocessing and Pretreatment

The GC-MS and LC-MS raw data form a two-dimensional array. Since this array is large, it is often compressed either by distributing spectra as a feature list or by providing only the total ion chromatogram (TIC) instead of the full array. All four participants that provided LC-MS data provided a feature list, and one of the two GC-MS labs provided their GC-MS TIC. Since the NMR spectra were only ^1^H 1D data, which is already a single array, the spectra were binned to reduce the effects of feature shifting from instrument to instrument. The preprocessing steps are demonstrated graphically in Figure 1A,B.

#### 2.4.2. Principal Components Analysis

The data were analyzed and scored using PCA [16] with bootstrap uncertainty analysis [17,18]. In this analysis, a set of random data arrays are generated by sampling from the original data array with replacement. PCA is then performed on those random arrays, and the original data array is transformed into these bootstrap PCA spaces. Because a PCA space is unique to within reflections about the coordinate axes, a Procrustes algorithm is used to align the PCA spaces [19]. Uncertainty in the PCA is quantified by fitting the bootstrap populations for each sample (e.g., all replicates of Sample A) to a multivariate normal distribution. Then, each participant’s measurements are scored by calculating their Hotelling T^2^ distances within the respective populations. This uncertainty analysis is presented graphically in Figure 1C. For a more complete description, see Appendix A.

#### 2.4.3. Principal Components Analysis on Feature List Data

The LC-MS data were provided as feature lists, and so it is not possible to perform PCA by simply combining all the data since each laboratory identified different numbers of features. To overcome this issue, we first conducted PCA on each participant’s data individually. Observing that all the PCA scores appeared visually similar to each other, to within rotations and reflections, we used a Procrustes algorithm to align the PCA scores using one lab’s PCA space as the basis. Since the labs scores results are similar, this choice was essentially irrelevant. We then transformed the Procrustes-aligned scores back into the basis lab’s feature list space, thereby creating a set of virtual feature lists that are representative of what those labs would have measured if they had been using the same instrument (see Appendix A for details of how the virtual feature lists are constructed). Since these virtual feature lists are now directly comparable, we performed the PCA uncertainty analysis on this whole set as described above.

## 3. Results

### 3.1. Overview

Table 1 shows the similarities of the three labs that performed NMR analysis. Since similarity comparisons in NMR are often based on PCA results, we define a similarity measure for PCA loadings spaces that is based on the cosine distance. The measure *d_ij_* between the loadings spaces of two labs, *i* and *j*, is calculated as

(1) dij=∑k12(σk,i+σk,j)(1−|pk,i⋅pk,j|)

where **p***_k,i_* and **p***_k,j_* are the *k*^th^ principal component in the PCA space of the *i*^th^ and *j*^th^ participants respectively and σ*_k,i_* and σ*_k,j_* are the explained variance ratio of the *k*^th^ principal component in those respective spaces. Note that this assumes that the PCA spaces are orthonormal, which is the case for the PCA algorithm used here. We use the absolute value of the dot product because PCs are unique to within factors of −1. This form results in a number that varies from 0, meaning two PCA spaces have the same PCs, to 1 if all corresponding PCs in the two spaces are orthogonal. We apply this analysis to the NMR data only, because the NMR bins are the same across all three participants and so the principal components are transformations of the same spectral feature space. Because the GC-MS and LC-MS data were reported in the form of feature lists, the spectral feature space is different for each lab.

As shown in Table 1, Labs 1 and 3 are quite close to each other at a PCA similarity distance of ~0.05, while Lab 2 is somewhat farther from these two at ~0.15. With only three data sets, it is impossible to quantitatively state what constitutes “close” or “far” in this sense. However, for vectors of large dimension such as the NMR spectra used, most vectors are orthogonal. Therefore, any distance less than 1 indicates that the PCA spaces are reasonably close. Furthermore, examination of the PCA scores indicates that all the reported data sets separate the samples similarly.

Table 2 shows the number of extracted ions that were extracted from the basic LC-MS experiments, the number of extracted ions with intensity RSD less than 20% across all samples that were replicated, and the overlap in extracted ions between participants. The mass tolerance was set to 30 ppm and retention time tolerance to 0.3 min. There was a great disparity between the molecular feature coverages of the different participants, thus the creation of metabolite signatures that are transferable across different instrumentation [11] or sample preparation protocols [10] is not a trivial matter. Even using overlapping molecular features having approximately same *m*/*z* values and retention times the uncertainty of such comparisons is high since most features are unidentified. 

Table 3 briefly summarizes the findings of the two labs that performed GC-MS characterizations on the derivatized extracts of the samples. Although the total number of peaks detected differed significantly between the two participants, probably based on peak detection thresholds, the number of peaks identified was quite similar and the identifications in-common represented 24% to 30% of the total number of peaks identified by the two labs.

The standardization and harmonization of comparisons across participants thus demands new strategies that address these complex issues while causing minimum disruption to the usual workflows. The appropriate use of certified reference materials, spectral libraries [20,21], and hybrid searches [22,23,24] may be a reasonable approach for these purposes. 

### 3.2. Detailed Results

#### 3.2.1. Nuclear Magnetic Resonance (NMR)

**Figure 2:**
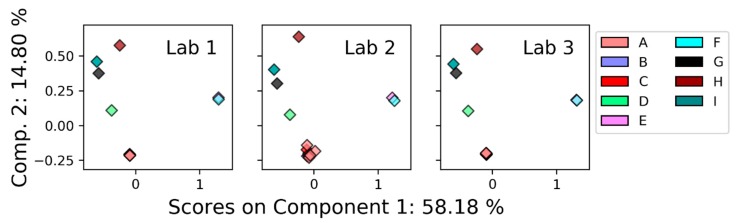
PCA scores for the three labs reporting NMR data.

All the labs were found to separate the samples in a similar fashion, as evidenced by the PCA scores shown in Figure 2. The primary difference between Lab 2 and Labs 1 and 3 is the variability in the spectra for Sample A from that Lab 2, which is likely also what introduces the difference in the PCA spaces shown in Table 1.

**Figure 3:**
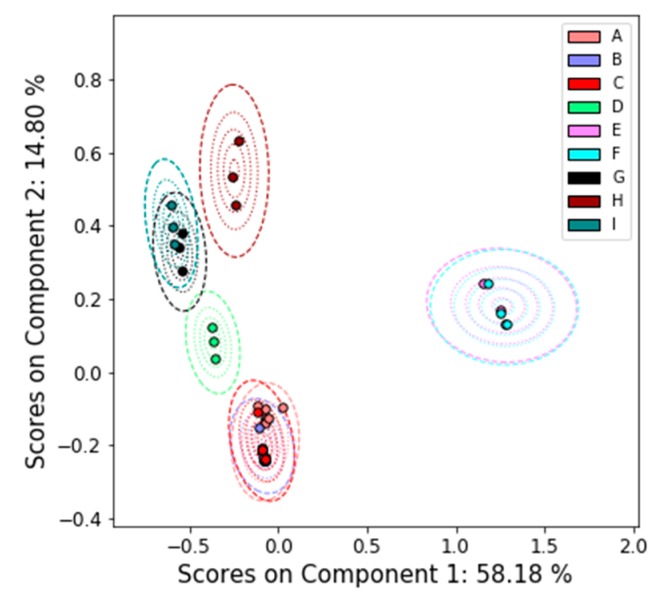
PCA results with uncertainty analysis on all of the ILS’s NMR data. The PCA scores are superimposed on contours of the T^2^ distance. Dotted contours correspond to the 5%, 25%, 50%, and 75% confidence limits. The dashed contour corresponds to the 95% confidence limit.

**Figure 4:**
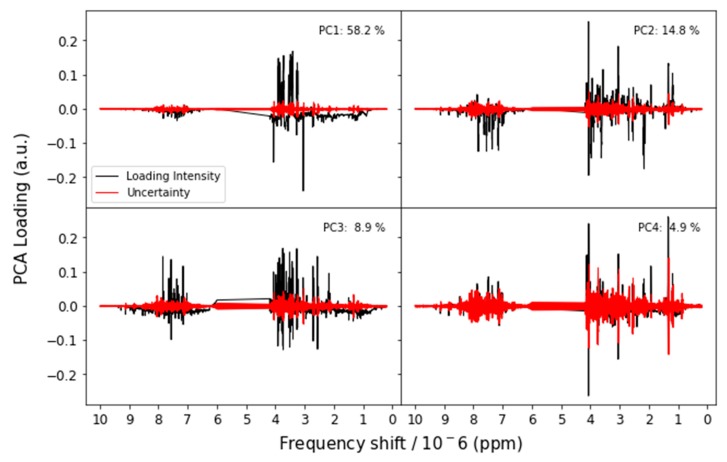
NMR PCA loadings with uncertainty. The uncertainty is drawn so that, if it obscures the NMR signal, the corresponding loading is not visible. Note that uncertainty is drawn as a symmetric interval around 0. There may appear to be a nonzero uncertainty in the solvent suppression region because uncertainty in the bins on either side may not be zero and there is no data in the suppression region; this apparent uncertainty is an artifact and should be ignored.

We estimate the uncertainty in the NMR spectra and the corresponding loadings by combining all the spectra into one data set and performing PCA on that combined set. This analysis confirms that the participants are all finding similar features, as evidenced by the PCA scores and T^2^ distances shown in Figure 3 and corresponding PC loadings shown in Figure 4. The loadings and explained variance of PC 1 also show that all the labs are identifying glucose and other sugars as the major difference among the samples. This suggests that samples E and F contained an unusually high proportion of high glucose contributions due to disease (such as diabetes) or uncontrolled dietary intake. The loadings of PC 2 are likely due to nicotine metabolites, as there is roughly a gradient along the PC 2 direction from nonsmokers to smokers.

Because this result includes only three participants, the T^2^ uncertainties in Figure 3 are very large compared to the actual scatter within the classes; the uncertainty is mostly controlled by the uncertainty in the spectra for Sample A from Lab 2. With more participants, the scatter in the scores would likely contribute more to the uncertainty of each sample.

Compound identifications and 2D JRES spectra were an optional portion of the ILS; only one participant reported these results. As such, we do not report on the compound identifications from that participant because we cannot compare it to other labs in the ILS as we can on the GC-MS and LC-MS platforms. Compound identification using NMR is less dependent on libraries and repositories than GC-MS and LC-MS. As such, NMR serves as a valuable tool for unknown compound identification [25].

#### 3.2.2. Liquid Chromatography–Mass Spectrometry (LC-MS)

As reported previously for plasma samples [11], results using the same LC-MS instrumentation and the same material are very repeatable. No more than five replicates are needed to find thousands of molecular features with intensity peak RSDs below 2% in batch to batch (intermediate precision) experiments. However, intensity fluctuations are more pronounced when comparing just slightly different matrices, such as the different urine samples in this work. Thus, in label-free untargeted experiments, the RSD threshold must be higher (~20%) for an effective comparison including a significant number of ions.

Table 3 shows the number of molecular features (peaks) extracted from the raw data of four participants. Feature extraction was performed using XCMS online [26,27], with the optimized settings for each instrument suggested by the authors, except for Lab 4 that used vendor-specific software. The number of peaks was extremely variable across participants, revealing that LC-MS data contain many peaks with little or no relevance for the bioanalysis. These low-influence peaks include, but are not limited to, signals from redundant in-source fragments, background ions, contaminants or electrospray artifacts. Intensity peak fluctuations also reduce significantly the number of useful features. The RSDs shown in Table 2 were derived from the absolute peak intensities, but even after normalization, many low abundance components showed significant deviations. More importantly, for interlaboratory comparisons, the only features that matter are those features found by all participants. Table 2 shows that in this ILS the comparison is limited by the overlapping of 1141 peaks between Lab2 and Lab4. Approximately 31% of these peaks were putatively identified using the NIST tandem mass spectral library (http://chemdata.nist.gov/mass-spc/msms-search/), which are shown in Table S1, Interlab_Table_SI_1.xlsx of the supporting information. Still, all participants were able to differentiate the urine matrices based on PCA analysis. The sample clustering and the principal component contributions were similar for all labs.

**Figure 5:**
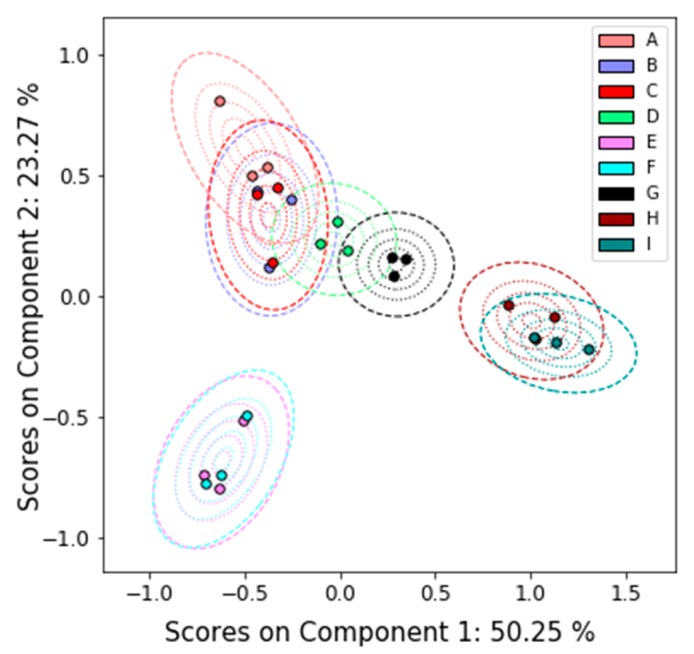
PCA results with uncertainty analysis on the ILS’s LC-MS data using virtual peak lists. Lab 4 is excluded from this analysis because of the relatively low number of features. Note that each sample (A–I) has three scores, one for each lab.

As discussed above, in the case of LC-MS, several labs provided feature lists, all of which are slightly different and depend on the threshold used for feature selection. This motivated our PCA on the virtual peak lists. We present the results of the PCA in Figure 5. In this case, much of the uncertainty is controlled by the E and F measurements. Due to the low number of features compared to the other participants, data from Lab 4 is excluded from the virtual peak list analysis. However, this participant was also able to differentiate the samples.

**Figure 6:**
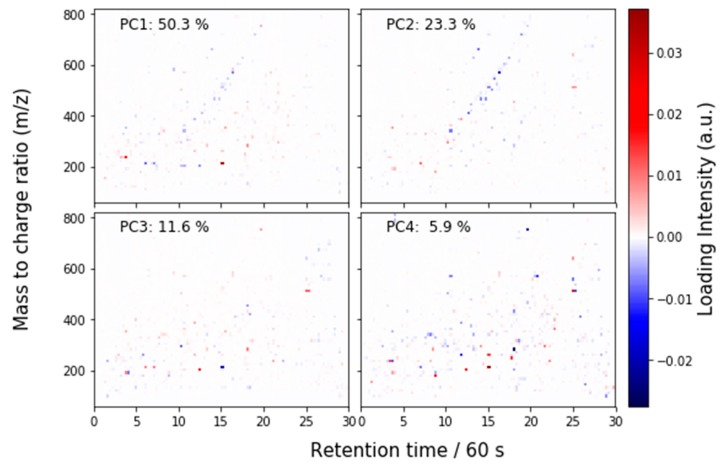
PCA loadings for the LC-MS data, interpreted as a chromatogram.

**Figure 7:**
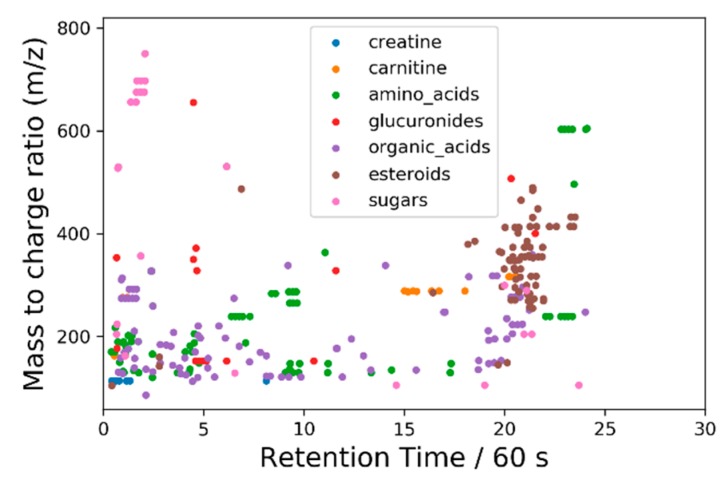
Chemical classes corresponding to selected LC-MS spectral features. This plot has the same scale as Figure 6.

Because the LC-MS analysis is performed on the full feature list, with *m*/*z* and retention time values, we can interpret the PCA loadings as full chromatograms. These are shown for the first four PCs in Figure 6. Some spectral features are identified by chemical class in Figure 7, which uses the same scale as Figure 6.

As expected, major contributions (loading intensities) come from compounds that ionize well in electrospray conditions, such as creatine, amino acids, multiple carnitine derivatives, and others, as shown in Figure 7. As mentioned before, a full list of identified compounds that are common to all labs is attached with the Supplemental Information (Interlab_Table_SI_1_mod.xlsx). These are considered putative identifications and are determined by the results of the high-resolution instruments and, if possible, confirmed by masses, retention times and fragmentation for the other labs. For example, one participant used a low-resolution instrument and did not perform MS^2^ experiments. Another participant used a different kind of fragmentation instead of MS^2^ experiments.

Even following a strict experimental protocol, results indicate significant differences among labs. In general, major fluctuations were found in the number of peaks and peak intensities reported from different labs. The lack of reproducibility mostly originated from low-abundance components that, depending on the ionization efficiency, can (or cannot) be detected consistently in a particular instrument. This illustrates how researchers should be cautious when using global untargeted experiments to suggest metabolite signatures for diseases or for monitoring metabolic changes. However, the fact that all labs were able to distinguish all samples and a substantial number of peaks were detected in all instruments shows that such inter-comparisons are possible if a method for peak identity conformation is available. There is no need for authentic identifications in these comparisons, only a putative ID, preferably based on the fragmentation spectrum, to connect samples in an unequivocal manner [11,20].

#### 3.2.3. Gas chromatography/Mass Spectrometry (GC-MS)

One of the labs reported a significant number of species attributable to the process/preparation blank; the most prominent peaks were tentatively identified as TMS derivatives of sugars, eluting between 47 min and 50 min. For this lab, samples B and I-2 did not yield derivatized extracts that were comparable to the other replicate preparations for these same samples in this ILS so their results were considered as outliers and were not included in the data analysis portion of the work.

**Figure 8:**
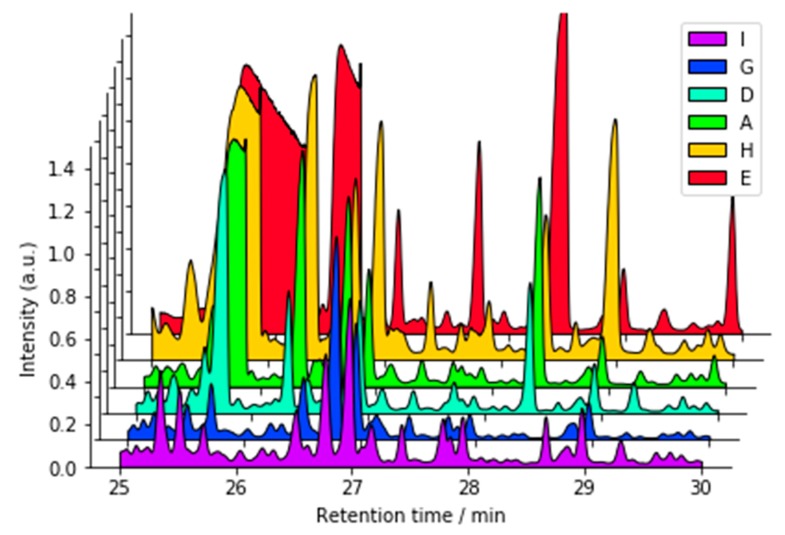
GC-MS total ion chromatograms.

In general terms, both participants that returned GC-MS data reported dozens of TMS derivatives of sugars, sugar alcohols, amino acids and metabolites of nutritive species (e.g., fatty acids) present in the extracts of the urines; identifications are shown in Tables S2–S8, Interlab_Table_SI_2.docx in the supporting information. One significant difference among the analyte peak profiles of the samples were the high levels of glucose TMS derivatives tentatively identified in samples A, E, and H. In the TIC shown in Figure 8, six peaks tentatively identified as glucose TMS derivatives in these three samples showed obvious signs of overloading the GC column. The other participant did not submit a chromatogram, but the reported level of glucose-related species was similar, suggesting that they also experienced overload. The usual procedure with overload is to dilute the samples by a large factor; however, this would have required restarting this portion of the ILS. Furthermore, the semi-quantitative approach employed here for compound identification remains valid despite the overloading. Previous work by Pasikanti and co-workers [28] describe urinary metabolite profiling using GC × GC with time-of-flight MS and a method similar to that described in this work. Struck-Lewicka et al. [29] performed both GC-MS and LC-MS urine metabolite characterizations as part of a prostate cancer study, and observed a similar suite of compounds.

**Figure 9:**
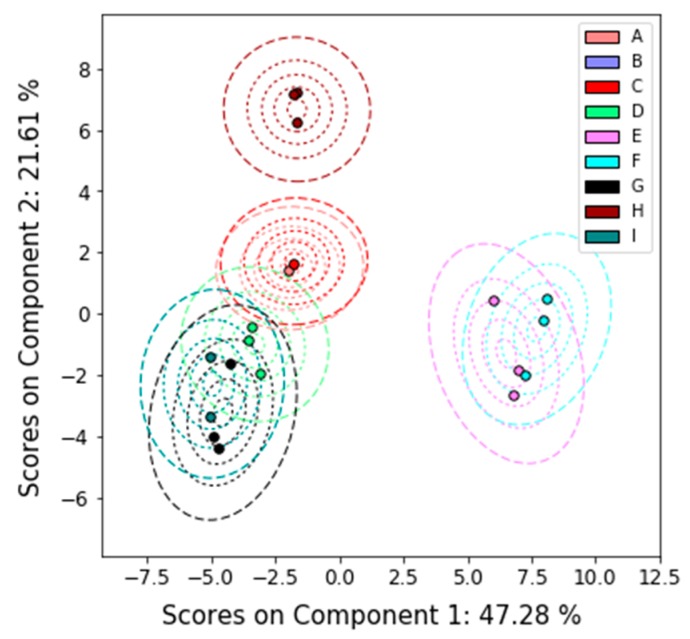
PCA results with uncertainty analysis on the ILS’s GC-MS data.

Similarly for the NMR data, we can conduct the uncertainty analysis on the GC-MS data. However, unlike the NMR data, it is not conventional to share raw GC-MS spectra, and so Figure 9 presents the results of an analysis using the data provided by the participant that shared the raw profiles. Since there is only one participant it is more difficult to draw conclusions. For instance, there is only one measurement for each of samples A and C, which therefore lie very close to the center of their respective bootstrap distributions, and so the corresponding T^2^ scores for those measurements are very close to 1. However, this participant obtained enough replicates to allow the determination of some estimates of the uncertainty in the GC-MS measurements.

**Figure 10:**
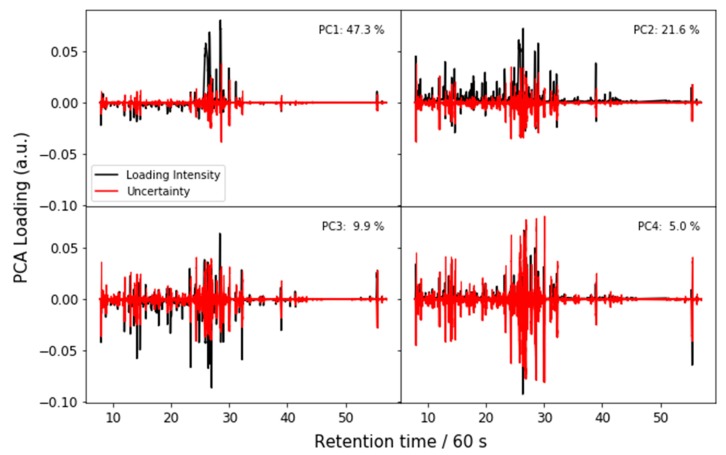
PCA loadings for the GC-MS total ion chromatograms. The interesting part is the sugar-relevant region from 20–35 min retention time.

The loadings for the first four PCs are shown in Figure 10. The major loadings for the first principle component are attributable to glucose derivatives, two unknowns (retention times of 27.8 min and 30.0 min) and a methylxanthine possibly from purine degradation. For the second principle component, histidine, galactose and mannitol derivatives provide the most significant loadings.

**Figure 11:**
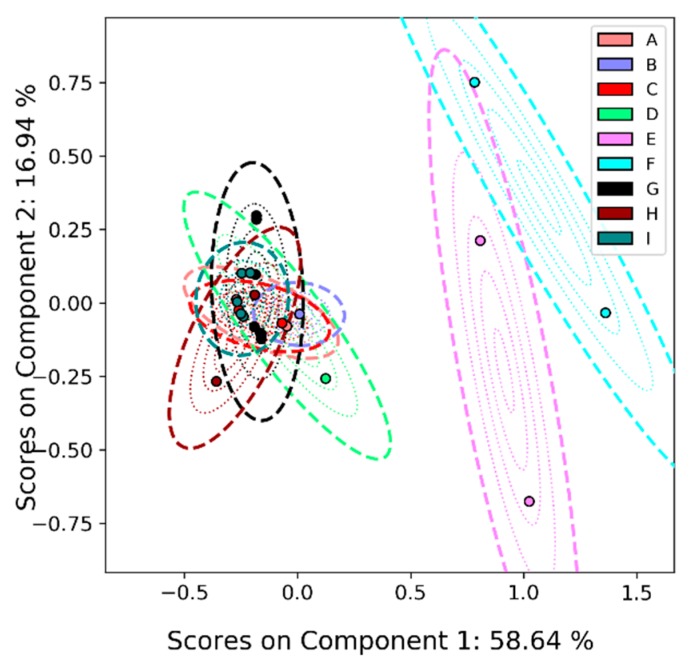
PCA results with uncertainty analysis on the ILS’s GC-MS data using virtual peak lists.

As it is difficult to draw conclusions from the data obtained from a single lab, we also performed the same virtual-peak analysis on the GC-MS data as on the LC-MS data, the results of which are shown in Figure 11. Unlike the LC-MS case, where the reconstructed spectra fell cleanly into distinct groups, in this analysis we see the samples split into one group consisting of samples E and F and another consisting of all other samples. We believe this is partly due to chemical differences between these two groups overwhelming any differences within the groups in the PCA. Even so, both labs still show the stark differences between these two groups, just as was observed for the other platforms.

#### 3.2.4. Data Dissemination

**Figure 12:**
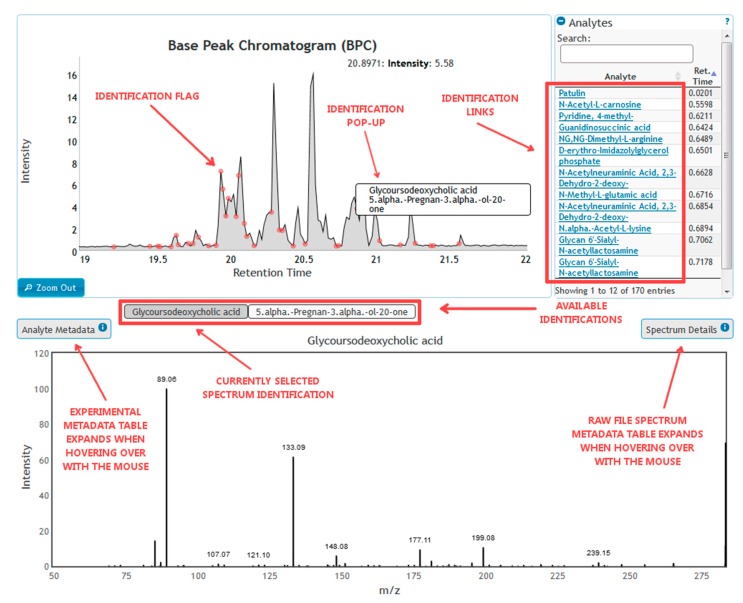
vMS-Share screenshot. This screenshot shows the program in use to analyze an LC-MS chromatogram in the vMS-Share experimental database. The figure on the top left shows the base peak chromatogram (or total ion chromatogram), with identified features flagged with red dots. To the right of the base peak chromatogram is the list of substances associated with each identified feature. In this use case, the user has hovered the pointer over one of the identification flags, which results in a pop-up with the species associated with that feature. Clicking on the flag has brought up the mass spectrum from the raw file for the identified chemical species at the bottom of the figure. There are two substances at this retention time, and the user can pick between the two.

GC and LC/MS data obtained from the ILS will be distributed using NIST’s Visual Mass-Spec Share (vMS-Share) website (vmsshare.nist.gov) [30]. vMS-Share is a mass spectrometry (MS) data mining and repository web site hosted by the NIST’s Chemical Sciences Division. The purpose of the web site is to effectively visualize raw MS data, display corresponding metadata and share experimental results. A screenshot of the web site, being used to investigate a chromatogram from the experimental data set, is shown in Figure 12 [30].

vMS-share currently serves as a repository for proteomic and metabolomic MS data that has been published in scientific journals. Plans are also being made to add petroleomic and SRM data in the future. The web site has the capability for performing study-based and experiment-based data mining/analysis search. Each search begins by first selecting a single study for data mining.

Experiment-based search is performed by picking a dataset of interest and selecting an experiment to view. An interactive graphical interpretation of the Base Peak Chromatogram with peaks containing identifications labeled on the chromatogram represents the selected experiment. A searchable list of identifications is also displayed along with the chromatogram for access to identified spectra. Identified and unidentified MS1 and/or MS2 visual representation of spectra can also be interactively accessed from the chromatogram viewer. Metadata associated with a spectrum from the raw file is displayed alongside the spectrum as well as any experimental identification metadata where available.

Study-based search is performed by searching for identifications within a selected dataset using a variety of easily accessible search filters such as CAS and protein accession numbers. Interactive spectrum display, raw metadata, and experiment metadata can be viewed by selecting a specific identification. Each identification is linked to the experiment that it belongs to and the entire experiment data associated with the selected identification can then be viewed.

## 4. Discussion

### 4.1. Test Materials as A Reference Suite

We have proposed a new approach for using test materials as quality control in *-omics* measurements. In our approach, we have compiled a suite of urine materials that can be used across multiple platforms; here, we studied nuclear magnetic resonance (NMR) spectroscopy and liquid and gas chromatography with mass spectroscopy (LC-MS and GC-MS). The materials were chosen to have subtle compositional differences that we believed would be detectable using these platforms, and we found that all platforms were able to distinguish among the test materials, even the lower-resolution platforms. The differences were generally consistent across different facilities employing the same technique, and sufficiently consistent across techniques to enable comparison between them. This shows that a suite of materials, suitably chosen, can be used for quality control purposes applied to omics studies. Furthermore, our study shows the value of having a suite of stable reference materials designated for this purpose, maintained in a central repository such as a national metrology institute.

### 4.2. Comparison between Platforms

Each platform has its advantages and disadvantages, and it is useful to identify whether these strengths are shared among the platforms or whether they complement each other. We found that all platforms were able to identify the differences between the various samples. Two of the samples were the same except that one had been intentionally spiked with a chemical marker that was not visible to these three platforms, and all three platforms grouped these samples together. Additionally, all platforms clearly identified that samples E and F contained some unusual metabolite concentrations. In NMR and GC-MS, these metabolites clearly include sugars such as glucose, as evidenced by the PCA loadings in Figure 4 and Figure 10. It is possible, therefore, that the urine pools for these samples contain an abnormally high fraction of donors with diabetes or some other dietary intake abnormality. In reversed-phase LC-MS, small sugars such as glucose elute very early with highly soluble compounds, and all sugars suffer from in-source fragmentation which makes them difficult to detect using this method. In spite of this issue, the LC-MS results show separation between samples E and F and the other urine samples. This may be due to other potential biomarkers associated with diabetes or sample matrix effects. Since all three platforms find the same difference, but for different reasons, they provide complementary information.

Furthermore, samples A through G are from populations not exposed to nicotine, while samples H and I are from populations exposed to nicotine from various sources. LC-MS identifies this trend, making PC 1 essentially a nicotine-exposure axis. NMR and GC-MS do not pick up this same trend, since for both platforms non-exposed sample G is mixed with exposed sample I (and GC-MS further mixed non-exposed D into this group). In this case, NMR and GC-MS are less useful than LC-MS for detecting this effect. The nicotine is an exogenous compound and not really in the same class as the endogenous metabolites normally of interest in metabolomics. However, in a properly controlled study, these platforms should be able to detect the exposure through biochemical effects related to the exposure without needing to observe the toxicant. This is the principle behind environmental metabolomics studies where the interest is in the mode of action of a toxicant, not the toxicant dose (which is known or estimated a priori).

As this study was underway, another interlaboratory study was published [31]. The authors conducted two tests, one analyzing human urine either spiked or not with 32 metabolite standards and another comparing plasma of rats either supplemented or not with vitamin D. Both studies were conducted using NMR and MS techniques. They concluded that the overall results from different platforms share high similarity and therefore that untargeted analysis across participants using different instrumentation is possible. The authors also pointed out that the analysis of individual components should be explored further.

As shown above, we agree that untargeted metabolite profiles from different instrumentation and techniques show similar results and, in our case, allowed us to distinguish between seven different urine matrices. However, substantial differences were found when analyzing individual components. For example, the LC-MS analysis showed significant differences in ionization efficiencies for the top 100 most intense ions. Most participants differ not only in the magnitude of the ionization, but also two out of four participants deviate more than 20% regarding the identity of the 100 most intense ions. A complicating factor is the existence of multiple ions for each component, each one probably having a different ionization efficiency in a different ion source. However, the differences remain even considering the different contributions from all ions, and the best agreement is found only when all overlapping features are used in the analysis. This shows that the fusion of interlaboratory data is feasible, but the data analysis should be done under a concerted protocol. This may not be applicable to data analysis restricted to some specific chemical family that shares the same ionizing group(s). For example, Fiehn and collaborators [32] found that results from the analysis of fatty acids using nine different LC-MS platforms were highly comparable.

### 4.3. Prospects and Availability of Test Materials

This ILS has demonstrated cross-platform suitability for this set of urine samples for metabolomics quality control materials. The next step in production and public release of these materials into a final QC material would normally include a large-scale ILS integrating the lessons learned from the study. Since the paradigm for this type of QC material is so different from past SRM development schemes, NIST has decided to take an alternative approach to making these materials available to the public. Therefore, a limited number of sample sets will be made available to laboratories that will pledge to analyze them according to the established protocol and deliver data to NIST within a specific time frame. Any material left over from these measurements will be available to the laboratory for other uses, including alternative protocols.

The response to this offer will be key to gauging interest in QC materials, which leads to marketability assessments for this class of materials. With adequate responses from the community, including full analyses and suggestions for additional protocols, NIST will be able to make responsible decisions about the future production of QC materials.

### 4.4. Contact

More information about the data sets collected and access to the limited number of sample sets can be obtained by contacting the corresponding author.

## 5. Conclusions

In this work, we proposed and investigated a new approach to the use of test materials in -*omics* research. We compiled a suite of test materials taken from the Nationals Institute of Standards and Technology Standard Reference Material inventory and conducted an interlaboratory study in which participants obtained untargeted analytical profiles for these materials using a variety of common metabolomics platforms. Our goal was to determine whether the various platforms could detect the subtle differences between the materials, whether the information obtained was the same across platforms, and whether the participants in the study detected the same compositional differences. We found that all platforms were able to detect these differences and that the study participants were generally consistent with each other, although the different platforms did differentiate among the test materials for different reasons. We have shown, therefore, the value of using a suitably designed suite of stable reference materials to be used for measurement harmonization.

## Figures and Tables

**Table 1:** NMR Interlaboratory overlap.

Lab		Distance from Lab	
	1	2	3
1		0.150	0.046
2	0.150	-	0.169
3	0.046	0.169	-

**Table 2:** LC-MS Interlaboratory metabolite coverage and overlap.

Lab	Number of Extracted Ions (Intensity > 5000 Counts)	Extracted Ions (RSD < 20% in All Samples)	Overlapping Extracted Ions with Lab			
			1	2	3	4
1	28,818	13,867	-	12,511	8159	1225
2	42,781	12,563	12,511	-	7857	1141
3	56,656	10,332	8159	7857	-	3734
4	4947	4932	1225	1141	3734	-

**Table 3:** GC-MS Interlaboratory metabolite coverage and overlap.

Lab	Total Number of Peaks Detected	Number of Identified Peaks	Identifications Overlapping
1	71 to 130	50 to 93	12 to 26
2	227 to 268	62 to 95	

## Supplementary Materials

The following are available online at https://www.mdpi.com/2218-1989/9/11/270/s1, Table S1: Interlab_Table_SI_1.xlsx; Tables S2–S8: Interlab_Table_SI_2.docx; the anonymized data: gcms_data.xlsx, lcms_data.xlsx, and nmr_data.xlsx; and the associated computer codes: gcms_processor.ipynb, lcms_processor.ipynb, nmr_processor.ipynb, mlu_plot_utils.py, and plot_utils.py.

## Author Copyright Statement

This article is a contribution of the National Institute of Standards and Technology (NIST), an agency of the Federal Government. Pursuant to Title 17 United States Code Section 105, works of NIST employees are not subject to copyright protection in the United States. This article may be subject to foreign copyright.

## Appendix A. Uncertainty Analysis

### Appendix A.1. Principal Components Analysis with Bootstrap Uncertainty (PCA-Boot)

Principal components analysis can be described briefly through Equation (A1),

(A1) $$X=TP^{T}+E$$

where **X** is the array of spectral data with each row representing the spectrum from one sample, **T** is the projection of the spectral data (i.e., scores) onto the PCA space with basis **P** (i.e., loadings), and **E** is the array of residuals. Uncertainty is conducted using bootstrap with replacement. For each bootstrap sample, a random data matrix **X*** is generated, of the same size as **X** and whose rows are samples with replacement from the rows of **X**. A PCA model is generated for the **X*** array which results in a corresponding **T*** and **P***. The bootstrap scores array **T**_boot_ is calculated as

(A2) $$T_{\mathrm{boot}}=XP^{*}$$

Because PCA loadings are equivalent to within a sign change, an orthogonal Procrustes algorithm is used to align **T**_boot_ with **T**. Once all of the **T**_boot_ arrays are calculated, they are concatenated into a single array where each row represents one bootstrap representation of one sample. The class population sizes are then determined by fitting a Hoteling T^2^ distribution to the members of each class.

### Appendix A.2. PCA-Boot on LC-MS Peak List Data

Because the LC-MS data is provided only as a peak list, rather than a full spectrum, it is more complicated to calculate the uncertainty. In this case, PCA is used on the peak list from each facility, that is,

(A3) $$X_{i}=T_{i}P_{i}^{T}+E_{i}$$

where the subscript *i* refers to the data from each facility, X_i_ is the array of intensity data with feature retention times as columns and sample runs as rows, **T***_i_* is the scores for this data in PCA space with basis **P***_i_*, and **E***_i_* is the array of residuals. To generate a set of data that can be easily compared with each other, we choose one laboratory as the “basis” laboratory and then use a Procrustes algorithm to align all of the **T***_i_* arrays with the base array. Then we use the base facility’s loadings array to map the aligned scores **T***_i_*_,aligned_ into a set of virtual peak lists **X***_i_*_,virtual_ according to

(A4) $$X_{i,\mathrm{virtual}}=T_{i,\mathrm{aligned}}P_{j}^{T}$$

where the subscript *j* refers to the facility selected as the base facility, **P***_j_* is the PCA basis for facility *j* (from Equation (A3)), and **T***_i_*_,aligned_ is the scores array for facility *i* after alignment with the **T***_j_* array (the scores array for facility *j* from Equation (A3)). The virtual peak lists are concatenated into an **X**_virtual_ array and PCA with bootstrapping, as described above, is then conducted on this new array.

### Appendix A.3. PCA-Boot Detailed Procedure

- Sample randomly with replacement from the binned, normalized spectra in **X** (generate **X***)
- Fit a Pareto scaler to the bootstrap sample **X*** (NMR only)
- Conduct PCA on the scaled **X*** (generate **T*** and **P***)
- Scale **X** using the Pareto scaler (NMR only)
- Transform the scaled **X** into the **X*** PCA space (generate **T**_boot_)
- Align **T**_boot_ with **T**

### Appendix A.4. PCA-Boot on Peak Lists Detailed Procedure

- Conduct PCA on each facility’s peak list (calculate **T***_i_* and **P***_i_*)
- Choose a lab to use as the base lab (choose *j*)
- Align all **T***_i_* arrays with **T***_j_* (generate **T***_i_*_,aligned_)
- Map aligned scores into virtual peak lists (generate **X***_i_*_,virtual_)
- Conduct PCA and bootstrapping on concatenated virtual peak lists.
