# Supplementary material for: Metabolomics Test Materials for Quality Control: A Study of a Urine Materials Suite
Source: Metabolites. 2019 Nov 7;9(11):270. doi: 10.3390/metabo9110270 (PMC6918257; doi:10.3390/metabo9110270)
Supplement: Supplementary file 1 [file metabolites-09-00270-s001.zip › supplementary materials/Interlab_2016_Protocols_Annex_final.pdf]

qMet 2016 Interlaboratory Comparison:
Protocols Annex

The following Protocols Annex contains guidance for the urine sample receipt and storage, as well as moderately constrained protocols for sample processing and analytical data generation and processing for the three key analytical platforms: NMR, LC/MS and GC/MS.

The relevant sections are hyperlinked below:

Annex 0. Sample Receipt and Storage

Annex A. NMR Protocol

Annex B. LC/MS Protocol

Annex C. GC/MS Protocol

Annex 0. Sample Receipt and Storage

Step 1. Receipt and Storage of Urine Samples:

1. NIST will select nine frozen urine samples for analysis, labeled ***qMet 2016: Sample A*** through ***qMet 2016: Sample I.*** In addition to the urine samples, the following peripherals will be supplied:
    a. For laboratories participating in NMR analysis, a solution of D_2O NMR buffer will be supplied frozen in 4 ml aliquot(s), labeled *qMet 2016: Buffer Solution*. In addition a commercial temperature calibration solution will be supplied.
    b. For laboratories participating in LC/MS analysis, one (1) Agilent Poroshell 120 EC-C18, 2.1 × 100 mm, 2.7 µm LC column will be supplied. In addition, an internal standard solution will be supplied, labeled *qMet 2016: Internal Standard*.
    c. For laboratories participating in GC/MS analysis, a commercial solution of Grob Mix will be supplied.
2. Samples will be shipped frozen on dry ice. Upon receipt, assess the samples to make sure they are well frozen with excess dry ice in the shipping container. If the samples arrive unfrozen or the labels are not clearly legible, contact us immediately at qMet@nist.gov
3. Store the vials at -80 ℃ until ready to process.
4. Treat the urine samples as human clinical specimens, using appropriate precautions according to your local guidelines for handling human samples.

ADD MANIFEST TABLE HERE BRUCE

<u>Annex A. NMR Protocol</u>

NMR Exercise Design:

The exercise is divided into three segments. The first segment will include *mandatory protocols* for all participants. The second and third segment consist of a number of *optional protocols*, and will incorporate additional experiments or protocols that are designed to expand the depth and breadth of the study.

SEGMENT 1

Mandatory: Starting with a set of well-characterized, frozen **Urine** samples described in Annex 0, and prepared according to Appendix A (Step 0) and Appendix A (Step 1), analyze them using 1-D ^{1}H experiments as described in Appendix A (Step 2). There will be 9 samples sent to each lab, along with 4 ml buffer solution (0.1 M Phosphate D_2O Buffer, pH 7.3, with internal standard), used to prepare 16 NMR samples for analysis. The data is to be processed and analyzed according to the protocol in Appendix A (Step 3), and principal components analysis is to be conducted following the protocol in Appendix A (Step 4).

Procedure:

1. Thaw the samples (Appendix A (Step 0))
2. Prepare the samples for NMR (Appendix A (Step 1))
3. Collect 1-D NMR spectra (Appendix A (Step 2)) (If you intend to participate in the optional segments, you may be able to save time collecting additional spectra here, especially if you are using automation.)
4. Process the spectra (Appendix A (Step 3)), integrate the required features and calculate the signal-to-noise for each spectrum. Report the data in the spreadsheet (Tab "03 Integration and SN").
5. Complete the spreadsheet Tab "02 Procedure 1-D" to record experimental conditions.
6. Perform PCA analysis of the spectra (Appendix A (Step 4)). Report results for sample loadings and scores (Tab "04 PCA Results") in the spreadsheet.
7. Report binned spectra (Tab "D01 Urine Bins"), full loadings (Tab "D02 Urine Loads ") and full scores (Tab "D03 Urine Scores").
8. Insert the text for the 1-D pulse sequence used on the spreadsheet. (Tab "D07 Pulse Sequences")
9. Compress the raw data and processed spectra into a .zip file for delivery to the coordinators.

SEGMENT 2

<u>Optional: Application of 2-dimensional J-resolved (JRES) NMR spectroscopy:</u>

Utilizing the same **Urine** samples from Segment 1, participants will collect 2-D homonuclear ^{1}H JRES spectra for the samples according to the protocol in Appendix A (Step 5). Data should be processed according to the guidelines in Appendix A (Step 6). Principal components analysis is to be conducted on the 1-D skyline projections following the protocol in Appendix A (Step 4).

Procedure:

1. Use the samples from Segment 1.
2. Collect 2-D NMR spectra (Appendix A (Step 5))
3. Process the spectra (Appendix A (Step 6)).
4. Complete the spreadsheet Tab “05 Procedure JRES” to record experimental conditions.
5. Perform PCA analysis of the spectra (Appendix A (Step 4)). Report results for sample loadings and scores (Tab “06 OPTION JRES PCA Results”) in the spreadsheet.
6. Report binned spectra (Tab “D04 Urine Bins OPTIONAL”), full loadings (Tab “D05 Urine Loads OPTIONAL“) and full scores (Tab “D06 Urine Scores OPTIONAL”).
7. Insert the text for the 2-D pulse sequence used on the spreadsheet. (Tab “D07 Pulse Sequences”)
8. Compress the raw data and processed spectra into a .zip file for delivery to the coordinators.

SEGMENT 3

Optional: Identification of compounds using NMR spectroscopy:

Utilizing one urine sample (***qMet 2016: Sample A***), identify as many compounds in the sample as possible. Additional NMR experiments may be run (TOCSY, ^{13}C-HSQC, etc.), but no chromatographic separations or derivitization is requested for the sample. No quantitation (relative or absolute) is requested. Please rely on NMR data only.

Procedure:

1. Use the samples from Segment 1.
2. Utilize the previously collected 1-D and 2-D NMR spectra plus whatever additional NMR spectra that are reasonable for your effort.
3. Develop evidence for the listed compounds listed on the upper half of Tab "07 OPTION Compound ID" and complete the spreadsheet as thoroughly as possible. Not all compounds listed are necessarily in this sample or at levels that may be attainable in a reasonable amount of time.
4. Additional compounds that you develop evidence for may be added in the lower section of Tab "07 OPTION Compound ID".
5. Compress the raw data and processed spectra into a .zip file for delivery to the coordinators.

Appendices for NMR Protocol

Appendix A
Step 0 - Initial Urine Sample Handling:

1. The samples can be thawed and held in refrigeration (4 ℃) for up to two weeks with no discernable effect.
2. To prepare the samples in one session:
    a. Thaw the samples on ice for ≈ 3-4 hours.
        i. Thaw the NMR buffer along with the samples.
        ii. Do not adjust pH of the D_2O buffer solution.
    b. Set the samples on the bench at room temperature for about 30 minutes (no longer than 45 minutes) to allow them to come to room temperature.
    c. Samples may be warmed in hand if ice still appears in the vials. Do not heat with any other heat source.
    d. After coming to room temperature, vortex the vials to ensure the solutions are homogenous.
    e. Let vials sit for 5 minutes to reduce foam.
    f. Urine samples may be used for NMR sample preparation at this point.
3. Re-seal the sample vials by replacing the stopper and using Parafilm to close the seal.
4. Place unused sample material and buffer solutions into refrigeration (4 ℃). Do not refreeze the samples if additional analysis is anticipated.

Appendix A
Step 1 - Preparation of Urine Samples for NMR Analysis

1. To prepare the samples for NMR:
    a. Start with the thawed samples (Appendix A (Step 0))
    b. Carefully open the vials and buffer. Be careful to handle the metal caps properly to avoid cuts.
        i. Pre-label sixteen 1.5 ml Eppendorf centrifuge tubes.
        ii. Record masses on the provided worksheet – Tab “01 Sample Preparation” using a 5-place, calibrated digital balance. Weigh the 1.5 ml Eppendorf microcentrifuge tubes and record the values in the spreadsheet/worksheet.
        iii. Using a calibrated digital pipette, combine 400 μl of sample (record mass) and 200 μl of buffer (record mass) into the Eppendorf microcentrifuge tube. Change pipette tips between every wetting to avoid cross-contamination.
        iv. This specific volume ratio is required so that all laboratories use the same final concentrations.
    c. Vortex thoroughly (10 s to 20 s per sample); centrifuge using a benchtop centrifuge for 2 to 5 mins to move all liquid to the bottom of the tubes.
2. Transfer between 500 μL and 600 μL (depending on specific probe requirements) of supernatant to 5 mm NMR tube, add cap, and analyze each sample.

Appendix A
Step 2 - 1-D NMR acquisition

1. Prepare instrument for accurate temperature work (Once per protocol).
    a. Sample temperature = 298 K (24.85 °C)
        i. The temperature must be calibrated for this exercise using a d_4-methanol sample (99.8% D) (Cambridge Isotope Laboratories, Inc., DLM-7042-60mm) (if you do not have one, please contact Dan Bearden). If the system managers do not want to re-calibrate the instrument(s) you use, then use this calibration sample to adjust the 'setting' of your temperature control unit so that the ACTUAL, MEASURED temperature is 298K ± 0.1K. For example, you may have to 'set' your controller to 297.5K to achieve an 'actual, measured' temperature of 298K. For advice or help, please contact Dan Bearden (dan.bearden@nist.gov).
        ii. The calibration procedure is derived from (Findeisen et al. 2007), and uses a simple one-pulse ^{1}H experiment (no solvent suppression).
        iii. Using the d4-methanol sample and the function $T = -16.7467 \cdot (\delta\Delta)^2 - 52.5130 \cdot \delta\Delta + 419.1381$, where $\delta\Delta$ is the chemical shift difference (in ppm) between the CHD_2 and OH peaks in the spectrum, the temperature should be adjusted to 298K ± 0.1K. The target chemical shift difference is 1.5454 ppm ± 0.0008 ppm.
        iv. Measurements should be made with one transient after the sample has been equilibrated for more than 10 minutes. The spectrum should be processed with an exponential line broadening sufficient to smooth out any fine structure from coupling (typically 3 Hz or more). Use the instrument software to peak-pick the two resonances, and calculate the difference to 4 decimal places.
2. Optimize the instrument for maximum temperature stability and lock stability.
3. Load experimental study sample into magnet (allow at least 5 min for temperature equilibration, but this is instrument dependent so it may be longer on your instrument.).
4. Tune and match the probe – follow your standard practice and report on form.
5. Lock to D_2O.
6. Shim (FWHM of TSP peak must be < 2 Hz when no window function is used; there should also be a discernable dip between the central peak and the ^{29}Si satellites.).
7. Set receiver gain – follow your standard practice and report on form. **This will be held constant for all the samples in the set.**
8. 1D ^{1}H NMR spectroscopy with a gradient NOESY presat pulse sequence (e.g. noesygppr1d on Bruker; equivalent on Agilent/Varian/JEOLs) using a calibrated 90-degree hard pulse – follow your standard practice and report on form.

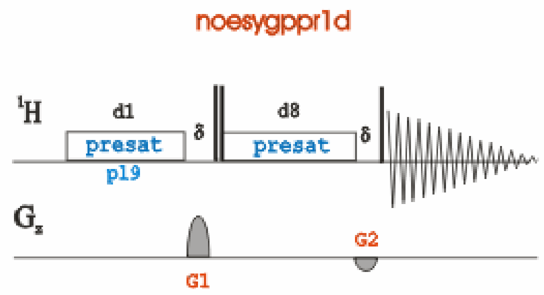

9. Use CW irradiation of water at low power during relaxation delay (equivalent to 10 Hz to 60 Hz irradiation which is reached with a power decrease of $-20\log(4 \cdot tau_h \cdot f_{cw})$ dB where tau_h is the duration in seconds of a high-power 90-degree pulse and f_{cw} is the desired irradiation width in Hertz) and during the NOE delay.
10. Optimize the mixing time (d8 in the above figure) for solvent suppression in the range of 0.01 s to 0.10 s.
11. 20.0 ppm spectral width, centered on the water resonance.
12. 3.0 s relaxation delay.
13. 80 transients (+8 dummy scans) for cryogenic probes; 160 transients (+8 dummy scans) for room temperature probes.
14. The acquisition time should be 2.0 to 3.3 s. For fields between 500 MHz and 800 MHz, this requires the acquisition of 64k real data points (e.g. TD=64k on Bruker; np=64k on Agilent/Varian).
15. Samples may be run in automation mode as long as the protocol above is followed for each sample.

Note: hardware (including NMR probe) and software used by participating lab must be declared, but we make no attempt to standardize on one platform only.

Appendix A
Step 3 - Post-acquisition processing [follow protocol for every spectrum]

1. Zero-fill once to 128k data points (e.g. SI = 64k on Bruker; fn=128k on Agilent/Varian).
2. Use an exponential line-broadening of 0.3 Hz.
3. No post-acquisition water suppression.
4. Fourier Transform the data.
5. Manual or Automatic zero and first order phasing.
6. Manual or Automatic baseline correction over the range 0 ppm to 10 ppm. The baseline correction protocol required is instrument dependent, so use your normal protocol and describe on the reporting form.
7. Calibration (TSP peak at 0.000 ppm).
8. Integrate the spectral features and the TSP singlet, using the spectral ranges defined in the spreadsheet (Tab “03 Integration and SN”).
9. Calculate the signal to noise ratio using your NMR analysis software with the signal (TSP) between 0.50 ppm and -0.50 ppm, and the noise region between 10.0 ppm and 11.0 ppm. (“sino real” command on Bruker)

Note: software used by participating lab must be declared, but we make no attempt to standardize on one platform only.

Appendix A
Step 4 - Spectral binning and principal components analysis [follow protocol for both Segment 1 and Optional Segment 2 1-D skyline projections.]

1. Bin/bucket start = 10.0 ppm.
2. Bin/bucket end = 0.2 ppm.
3. Bin/bucket size = 0.005 ppm. (This gives 1,960 buckets, total.)
4. Excluded regions:
    a. Urine Samples: water and urea (for example, 4.72 to 6.25 ppm).
    b. NOTE: These regions ***may*** be modified in the exercise spreadsheet and additional regions may be identified in the exercise spreadsheet; please check there for the authoritative values.
5. Bin all the collected spectra, except the blanks.
6. Normalize to total spectral area (Area = 1.0 is most common normalization value.).
7. No transformation or spectral alignment is allowed.
8. Compile the spectra into matrix. **PLEASE NOTE:** There are specific instructions on the spreadsheet regarding which samples to use for PCA.
9. Mean center the columns (some software packages do this automatically).
10. Perform principal components analysis (PCA) using Pareto weighted variables.
11. Export binned spectra with spectra as columns (so the data can be imported into Excel, for example), tab delimited, leaving in zeroed out bins. Also export scores and loadings as required in the spreadsheet.

Note: software used by participating lab must be declared, but we make no attempt to standardize on one platform only.

Appendix A
Step 5 – OPTIONAL Segment 2: 2-D JRES NMR acquisition

1. It is usually most convenient to conduct the JRES experiment immediately after the corresponding 1-D experiment; if this is the case, the sample should already be locked, shimmed, pulse calibrated, probe tuned and temperature stabilized.
2. Perform an absorption mode 2-D ^{1}H NMR JRES spectroscopy experiment with a gradient presat J-RES pulse sequence (e.g. jresgpprqf on Bruker; equivalent on Agilent/Varian) using a calibrated 90-degree hard pulse.

   Presat – 90 – delayTau – Gradient – 180 – Gradient – delayTau – Acquisition

3. CW irradiation of water at low power during relaxation delay (equivalent to 10 Hz to 60 Hz irradiation which is reached with a power decrease of $-20\log(4 \cdot tau_h \cdot f_{cw})$ dB where tau_h is the duration in seconds of a high-power 90-degree pulse and f_{cw} is the desired irradiation frequency in Hertz) and during the NOE delay.
4. 16 - 17 ppm spectral width in F2 (centered on water); 50 Hz spectral width in F1.
5. TD2 = 8k (8192) real points, TD1 = 32 increments.
6. 4.0 s relaxation delay.
7. 8 transients (+8 dummy scans) per increment for cryogenic probes (~20 minute experiment); 24 transients (+8 dummy scans) for room temperature probes (~60 minute experiment).

Note: hardware (including NMR probe) and software used by participating lab must be declared, but we make no attempt to standardize on one platform only.

Appendix A
Step 6 – OPTIONAL Segment 2: Processing 2-D JRES NMR data

1. Zero-fill to 16k complex data points (e.g. SI = 16k on Bruker; fn=32k on Agilent/Varian) in F2 and 128 complex data points (eg. SI = 128 on Bruker; fn = 256 on Agilent/Varian) in F1.
2. Apply spectral calibration in the F2 dimension equivalent to the corresponding 1-D spectrum. Apply spectral calibration in the F1 dimension so that the spectrum is centered at zero hertz in F1.
3. Use unshifted sine functions for apodization in both dimensions. (SSB 1 = SSB 2 = 0 on Bruker)
4. Fourier Transform the data in both dimensions for absorption mode presentation.
5. No phasing is necessary for absorption mode spectra.
6. Apply automated linear baseline correction in the F2 dimension.
7. Apply software 45-degree-tilt correction.
8. Apply automated fifth order baseline correction in the F1 dimension.
9. Symmetrize around a central horizontal line.
10. Calculate a skyline projection along F2 using rows 2 through 128 (row 1 usually has artifacts), and store this 1-D spectrum in an appropriate location.
11. The equivalent sequence of commands for Bruker Topspin software is:
    a. SR (enter appropriate values for spectral referencing)
    b. XFB
    c. ABS2 (ABSG_2=1)
    d. TILT
    e. ABS1 (ABSG_1=5)
    f. SYMJ
    g. F2PROJP(2,128,2) – Places the positive skyline projection from rows 2 through 128 into processed data file 2.

References for Annex A. NMR Protocol

Findeisen M, Brand T, Berger S. 2007. A 1H-NMR thermometer suitable for cryoprobes. Magn. Reson. Chem. 45:175.

Annex B. LC/MS Protocol

Workflow: Sample Preparation → Precipitation and/or Urine Dilution → Data Processing → Extract Chromatograms with 20 ppm mass accuracy → Custom Report

All experimental settings and data will be reported in an Excel spreadsheet named **qMet_2016_LC-MS_report**. The spreadsheet contains several sections (sheets), one to report the LC/MS settings and one for each sample to report extracted experimental results (sheets: SampleA_Info, SampleB_Info, …, SampleA_IDs).

Standard Sample Preparation:
Overview: Thawed urine sample → Methanol addition in ratio of 1:1 → Centrifugation → Lyophilization → Reconstitution

1. Centrifugation/precipitation and dilution. Prepare the urine samples as described below. Adjust quantities and instrument settings accordingly, depending on different vendor requirements.
   (a) Withdraw urine SRM samples from -80 ℃ and leave it for 3 hour at room temperature to thaw. Make sure that the urine SRM sample is completely thawed before you continue. Vortex the urine sample for 30 seconds.
   (b) Pipette 300 µl into a borosilicate glass vial (or a low-binding plastic vial) and add 300 µl of methanol. Cap the tube, vortex it for 30 seconds, and then centrifuge it for 20 min at 10,000g.
   (c) Move the supernatant to a new vial and remove the solvent by roto-evaporation at 25 ℃.
   (d) Reconstitute the dried samples in 200 µl of internal standard solution. Use glass or low-binding plastic vials for the analysis (*prepared sample can be stored at − 20 °C or below*).
2. Load vials or 96-well plate into autosampler maintained at 10 ℃.

Calibration Model and LC/MS method:

1. Carry out instrument setup i.e., (a) accurate mass and (b) calibration.

   (a) Accurate mass: Infuse appropriate concentration of leucine enkephalin (or alternative lockmass solution) into instrument. Follow set-up procedures.

   (b) Calibration: Infuse the usual proprietary calibration solution into the instrument. Follow setup procedures. The residual (in mDa) on each individual calibration point should be < 1.5 mDa. Ideally, the majority of calibration points will have residuals of < 0.5 mDa. Report residual errors. Adjust the optimum scan speed for your instrument (preferably higher than 10 spectra/second).

2. Follow the guidelines below to adjust the LC/MS conditions for the analysis.

Liquid chromatography conditions:

(a) Column: Agilent Poroshell 120 EC-C18, 2.1 × 100 mm, 2.7 μm (p/n 695775-902) or similar.
(b) Eluent A, 0.1% Formic acid aqueous solution; B, 0.1% Formic acid LC/MS grade acetonitrile solution
(c) Injection volume: 3 μL
(d) Flow rate: 0.4 mL/min
(e) Gradient (time table): {time (min), % B}: {(0, 1) (1, 1) (18, 20) (24, 90) (27, 90) (28, 1) (30, 1)}
(f) Column temperature: 35 ℃
(g) MS settings (only Agilent QTOF):
(h) Ion source: JetStream
(i) ESI Drying gas temperature and flow: 350 ℃, 8 L/min
(j) Nebulize 40 psi
(k) Ionization mode: Positive
(l) Capillary voltage: 3,500 V

The LC/MS settings may vary from instrument to instrument, so it is important to include details of the LC/MS setting of your platform in the LC/MS report. Specifics of the setting will be introduced manually in Sheet # 1 of the Excel spreadsheet, **qMet_2016_LC-MS_report.xlsx**.

LC/MS measurements:

1. Run all samples using a blank in between samples; a replicate of Sample A has been included as a ‘conditioning’ injection, this data will be discarded (Suggestion: Prepare a worklist following Table B-1 and use the suggested name for each experiment.) Run all blanks with the same method as the previous sample.

Table B-1. Worklist for Experiment 1.

Sample	Suggested name	Flow rate, mL/min	Injection volume, μL	MS levels	Metabolite profiling method
**A**	A	0.4	3	1	MS^1 Conditioning run
Blank	Blank1	0.4	3	1	
**A**	A	0.4	3	1	MS^1 (optimum scan speed)
Blank	Blank2	0.4	3	1	
**B**	B	0.4	3	1	MS^1 (optimum scan speed)
Blank	Blank3	0.4	3	1	
**B**	B	0.4	3	1-2	Untargeted profiling: 1 MS^1 scan followed by 4 MS^2 scans
Blank	Blank4	0.4	3	1	
**C**	C	0.4	3	1	MS^1 (optimum scan speed)
Blank	Blank5	0.4	3	1	
**D**	D	0.4	3	1	MS^1 (optimum scan speed)
Blank	Blank6	0.4	3	1	

**D**	D	0.4	3	1-2	MS^2 (Targeted list from MS1 results)
Blank	Blank7	0.4	3	1	
**E**	E	0.4	3	1	MS^1 (optimum scan speed)
Blank	Blank8	0.4	3	1	
**F**	F	0.4	3	1	MS^1 (optimum scan speed)
Blank	Blank9	0.4	3	1	
**G**	G	0.4	3	1	MS^1 (optimum scan speed)
Blank	Blank10	0.4	3	1	
**H**	H	0.4	3	1	MS^1 (optimum scan speed)
Blank	Blank11	0.4	3	1	
**I**	I	0.4	3	1	MS^1 (optimum scan speed)
**Blank**	Blank12	0.4	3	1	
**J**	J	0.4	3	1	MS^1 (optimum scan speed)
Blank	Blank13	0.4	3	1	
**K**	K	0.4	3	1	MS^1 (optimum scan speed)
Blank	Blank14	0.4	3	1	
**L**	L	0.4	3	1	MS^1 (optimum scan speed)
Blank	Blank15	0.4	3	1	
**M**	M	0.4	3	1	MS^1 (optimum scan speed)
Blank	Blank16	0.4	3	1	
**N**	N	0.4	3	1	MS^1 (optimum scan speed)
Blank	Blank17	0.4	3	1	

2. (Optional) Run sample **I** using a LC/MS method of your preference providing maximum metabolite coverage and identifications (targeted or untargeted analysis). Use any chromatography of your choice. The data from this analysis may be reported in any format, but it should include the basic information listed in the Excel spreadsheet, **qMet_2016_LC-MS_report.xlsx**. This data will not be released on the qMet website.

Data Processing: Use appropriate software to extract and align all mass signals above a defined threshold. A signal-to-noise threshold of 5 is appropriate. Labs with capabilities to carry out multivariate analysis, such as PCA, are encouraged to use it to report differences between samples and eliminate outliers. The output data must be transferred into MS Excel (file qMet_2016_LC-MS_report.xlsx) for basic statistical analysis or multivariate statistical analysis.. Convert your LC/MS data file (e.g. .raw, .yep, .d) to the open-source format mzXML by downloading and using the latest version of ProteoWizard's MS Convert http://proteowizard.sourceforge.net/ downloads.shtml. Upload the mzXML and raw files to the NIST website.

Criteria for assessing data quality (optional): The data obtained from the test (control) mixture (RT, peak shape, signal intensity, mass accuracy and so on) can be used to rapidly determine if the instrumental setup is suitable for the analysis. If major changes have occurred, this would automatically invalidate the results obtained for the samples.

QC sample assessment (optional): Use simple multivariate approach of PCA to help validate the analyses. The PCA should show that the first 'conditioning' injections of the QC sample 'track' towards the main group of QC samples as the analytical system equilibrates. After confirming that the system has been adequately stabilized, the data for these initial 'blank' injections of the QC samples are discarded and the data derived from the in-run QC samples can then be scrutinized as follows: b) Report retention time (3 decimals), mass (4 decimals), S/N, maximum intensity, and area for each molecular feature in each experiment in the corresponding sheet of the Excel spreadsheet, **qMet_2016_LC-MS_report.xlsx**.

Annex C. GC/MS Protocol

All experimental settings and data will be reported in an Excel spreadsheet named **qMet_2016 GC-MS_report.xlsx**. The spreadsheet contains several sections (sheets), one to report the GC/MS settings and one for each sample to report extracted experimental results (sheets: SampleA_Info, SampleB_Info, …, SampleA_IDs).

Sample preparation: Use the sample preparation procedure described by Chan and co-workers (Nature Protocols, 6, 1483-1499, 2011) processing each of the nine urine samples, samples qMet "G" and qMet "I" in triplicate, and at least one processed blank sample consisting of all the reagents used for the derivatization procedure without a urine sample:

1. Thaw urine samples at room temperature or on ice (if the samples were stored at -80 ℃).
2. Vortex mix each thawed urine sample for 1 min.
3. Aliquot 200 µl of each urine sample into individual 2 mL microcentrifuge tubes. Process any QC samples typically used in your lab in the same way as the qMet urine samples.
4. Randomize the aliquoted metabolomic study samples along with blanks and QC samples and label them accordingly.

● PAUSEPOINT- Aliquoted samples can be stored at -20 ℃ for a month prior to subsequent processing.

Depletion of urea and sample extraction:

5. Add 20 µL of urease suspension (equivalent to 100 U of urease; refer to "*Reagent Preparation Instructions*" below) to 200 µL of each urine sample and incubate at 37 ℃ for 1 h to decompose and remove excess urea. *CRITICAL STEP:* Mix the urease suspension frequently to avoid variable addition of urease enzyme due to sedimentation of the enzyme.
6. Add 1.7 mL of methanol to the mixture and vortex-mix at high speed for 5 min at room temperature to precipitate urease enzyme and extract urine metabolites
7. Centrifuge all the samples subsequently for 10 min at 10,000 g and 4 ℃.
8. Carefully separate 1.5 ml of each supernatant into glass tubes and evaporate to dryness at 50 ℃ under a gentle stream of nitrogen gas using the TurboVap LV or similar device. Typically, 30–45 min is required for drying the supernatant.

● PAUSEPOINT- Dried samples can be stored at − 20 °C or below for up to 1 week.

10. Add 100 µL of toluene (dried over anhydrous sodium sulfate, refer to "*Reagent Preparation Instructions*") to the dry residue, mix for 1 min and dry again at 50 °C under nitrogen gas for 20 min. *CRITICAL STEP:* Avoiding this step could result in reproducibility issues because the derivatizing efficiency of MSTFA decreases with the presence of moisture in the sample. Traces of moisture can be removed by toluene, which forms an azeotrope with water. This drying step may be repeated if there is any doubt about the dryness of the sample. (Estimated time to completion the sample derivatization ≈ 3.5 h)
11. To the dried metabolic extract add 50 µL of MOX reagent (methoxylamine, 20 mg/mL; refer to "*Reagent Preparation Instructions*") and incubate the samples in the oven at 60 ℃ for 2

h. <u>CAUTION</u>: *Methoxylamine is highly toxic and an irritant to skin*. The derivatization should be performed in a fume hood. Temperatures higher than 60 °C may require special type of glass tubes to withstand high pressure. <u>*CRITICAL STEP*</u>*:* Glass tubes should be capped immediately to avoid absorption of moisture from the environment.

12. To each sample, rapidly add 100 µL of MSTFA with 1% (vol/vol) TCMS and incubate the mixture for 1 h at 60 °C to form TMS derivatives. <u>*CAUTION*</u>*: MSTFA is highly toxic. Therefore, derivatization should be performed in a fume hood.* <u>*CRITICAL STEP*</u>*:* Glass tubes should be capped rapidly to avoid degradation of both MSTFA and the derivatized metabolites.
13. Allow TMS derivatives to cool and transfer 100 µL of supernatant into GC vials. <u>*CRITICAL STEP*</u>*:* The derivatized samples should be analyzed immediately whenever possible. Derivatized samples can be stored up to 24 h at room temperature, but storage in a freezer (-20 °C) is recommended.
14. Preparation of commercial Grob Mix sample: For split injection, dilute the Grob Mix sample by a factor of 10 (v/v, e.g., 100 µL diluted to 1 mL) with dichloromethane. For splitless or on-column injection, dilute Grob Mix by a factor of 100 (v/v, e.g., 10 µL diluted to 1 mL) with dichloromethane.

<u>*Reagent Preparation Instructions:*</u>

- Urea Suspension: add 160 mg of urease of Sigma type III enzyme to 1 mL of Milli-Q water (this should provide enough suspension to process approximately 50 samples).
- Methoxyamine HCl in pyridine: dissolve 20 mg methoxyamine HCl in 1 mL pyridine.
- Drying of toluene: add sodium sulfate to toluene until no visible lumps are formed. Shake for 10 min and allow sodium sulfate salt to settle to the bottom of solvent bottle. Separate the supernatant toluene and store under dry conditions (up to 3 d).

GC/MS Measurements: Participants should measure their typical suite of targeted and non-targeted species and report relevant standards and calibration data used in quantifying the targeted compounds, as well as the mass spectral databases used for (tentative) identifications of the non-targeted components detected in the samples.

Participants are encouraged to use GC columns with phases similar to the bonded methyl polysiloxane, such as DB-1 (Agilent J&W Scientific), Rtx-1 (Restek Corp), or ZB-1 (Phenomenex Inc.) or 5% phenylmethyl-substituted polysiloxane such as DB-5, Rtx-5, or ZB-5 as examples, with dimensions similar to 30 m or 60 m × 0.25 mm (internal diameter) × 0.25 µm (phase thickness). If other GC column phases are used, they should be specified with as much detail as available. Participants are also encouraged to operate the columns at optimized linear velocities in the range of 30 to 40 cm/s with constant helium flow. The GC temperature program should be specified in the data reporting spreadsheet and could be similar to the following: 130 °C for a 1 min hold then a 5 °C/ min ramp to 300 °C for a 10 min hold at this final temperature. The MS should be scanned from mass/charge (m/z) 40 to m/z 600. It is recommended that the MS be autotuned prior to beginning the sample characterizations. If multi-day sequences are performed, autotune the MS prior to running each sequence. The running of the Grob Test mixture should be performed three times during the sequence(s) at the beginning, middle, and

towards the end of the sequence(s). Results from measurement of the Grob Mix are useful for evaluating chromatographic resolution, injector/column inertness, and mass spectral calibration.

Table C-1. Possible Sequence for Measuring Derivatized Urine Samples, Blanks, and Grob Test Mixture by GC/MS

Sample	Suggested name	Injection volume, μL
**A**	A	1
Blank	Blank1	1
**B**	B	1
Blank	Blank2	1
**C**	C	1
Blank	Blank3	1
**Grob Mix**	GrobMix1	1
Blank	Blank4	1
**D**	D	1
Blank	Blank5	1
**E**	E	1
Blank	Blank6	1
**I-3**	I3	1
Blank	Blank7	1
**G-2**	G2	1
Blank	Blank8	1
**F**	F	1
Blank	Blank9	1
**G**	G	1
Blank	Blank10	1
**I-2**	I2	1
Blank	Blank11	1
**G**	G	1
**Blank**	Blank12	1
**Grob Mix**	GrobMix2	1
Blank	Blank13	1
**G-3**	G3	1
Blank	Blank14	1
**I-3**	I3	1
Blank	Blank15	1
**Grob Mix**	GrobMix3	1

Data Processing: In addition to quantifying targeted species typically measured in your laboratory in the processed urine samples (e.g., creatinine), participants should endeavor to identify as many non-targeted species as possible with relative abundances in the total ion chromatograms (TICs) of 10% or greater. Processing should involve background subtractions of mass spectra of selected chromatographic peaks followed by searches of relevant MS libraries

(e.g., NIST, Wiley). Targeted and non-targeted peaks processed for quantitative measurements and qualitative (tentative) identification should be noted by mean concentrations units and uncertainties, retention times, selected ions used for quantification, tentative identifications and related match scores from library searches, as appropriate.

Data Formats: If at all possible, convert raw data files to txt files using Proteo Wizard MSConvert. This conversion software should work for newer Agilent *.D files, including those acquired using MassHunter software. MSConvert should also work for other data acquisition software including ThermoFisher and Leco. Header files containing all instrument method parameters can be converted to a *.doc file and uploaded.
