# Supplementary material for: Metabolomics Test Materials for Quality Control: A Study of a Urine Materials Suite
Source: Metabolites. 2019 Nov 7;9(11):270. doi: 10.3390/metabo9110270 (PMC6918257; doi:10.3390/metabo9110270)
Supplement: Supplementary file 1 [file metabolites-09-00270-s001.zip › supplementary materials/Interlab_Table_SI_2.pdf]

Table S2. Tentative Peak Identifications for GC/MS of SRM 3667 Creatinine in Frozen Human Urine

Peak #	Retention Time (min)	Scan number	Peak Area	Tentative Identification	Match Score
1	7.95	841	77506279	Glycine, 3TMS derivative	920
2	8.035	867	16248724	Butanedioic acid, 2TMS derivative	973
3	8.274	937	46959124	Glyceric acid, 3TMS derivative	969
4	8.561	1021	31924853	(R*,R*)-2,3-Dihydroxybutanoic acid, tris(trimethylsilyl)-	954
5	8.775	1083	56109336	R*,R*)-2,3-Dihydroxybutanoic acid, tris(trimethylsilyl)-	963
6	8.978	1143	23983222	Serine, 3TMS derivative	924
7	10.215	1508	15038993	4-Methoxy-2-[(trimethylsilyl)oxy]phenyl}{2-[(trimethylsilyl)oxy]phenyl}methanon	762
8	10.281	1527	20924507	Butanoic acid, 2,4-bis[(trimethylsilyl)oxy]-, trimethylsilyl ester	925
9	10.82	1685	42703023	Butanoic acid, 3,4-bis[(trimethylsilyl)oxy]-, trimethylsilyl ester	951
10	11.116	1771	44073604	????	
11	11.956	2019	46295770	Aminomalonic acid, tris(trimethylsilyl)-	933
12	12.72	2244	42916312	meso-Erythritol, 4TMS derivative	939
13	12.981	2321	213761026	meso-Erythritol, 4TMS derivative	954
14	13.257	2402	20223178	Salicylic acid, 2TMS derivative	906
15	13.6	2502	62630709	L-5-Oxoproline, , 2TMS derivative	950
16	13.986	2616	350987490	????	
17	14.096	2648	174508721	L-Threonic acid, tris(trimethylsilyl) ether, trimethylsilyl ester	936
18	14.605	2798	175659506	L-Threonic acid, tris(trimethylsilyl) ether, trimethylsilyl ester	828
19	15.176	2965	42336481	Sebacic acid, di(4-octyl) ester	839
20	15.651	3106	88667492	1,2-Dithiane-4,5-diol, (Z)-, 2TMS derivative	945
21	16.488	3352	30467457	????	
22	16.826	3451	19966999	????	
23	17.137	3542	126251068	Tartaric acid, 4TMS derivative	920
24	17.378	3614	36125871	4-Hydroxybenzeneacetic acid, 2TMS derivative	923
25	17.516	3653	20634001	2-Butenedioic acid, (E)-, 2TMS derivative	860
26	17.726	3715	36704913	D-Arabinose, tetrakis(trimethylsilyl) ether, ethyloxime (isomer 2)	903
27	17.847	3751	123902002	3,4,5-Trihydroxypentanoic acid, tetrakis(trimethylsilyl)-	938
28	17.955	3783	75036175	DL-Arabinose, tetrakis(trimethylsilyl) ether, methyloxime	895

Peak #	Retention Time (min)	Scan number	Peak Area	Tentative Identification	Match Score
30	18.431	3922	35782188	D-Arabinose, tetrakis(trimethylsilyl) ether, ethyloxime (isomer 1)	828
31	19.246	4162	23285216	????	
32	19.339	4190	110051963	Levoglucosan, 3TMS derivative	924
33	19.856	4342	109951628	D-(+)-Arabitol, 5TMS derivative	957
34	20.022	4382	63235257	L-Fucitol, 5TMS derivative	831
35	20.96	4667	81586284	Aconitic acid, (Z)-, 3TMS derivative	950
36	21.321	4772	49574602	Ribonic acid, 2,3,4,5-tetrakis-O-(trimethylsilyl)-, trimethylsilyl ester	903
37	21.647	4867	21331946	Ribonic acid, 2,3,4,5-tetrakis-O-(trimethylsilyl)-, trimethylsilyl ester	867
38	21.921	4948	93084470	-(-)-Tagatose, pentakis(trimethylsilyl) ether, methyloxime (anti)	783
39	22.357	5079	17481598	????	
40	22.789	5205	20213817	9H-Purin-6-ol, 2TMS derivative	807
41	23.011	5270	48462005	????	
42	23.302	5325	714280064	Citric acid, 4TMS derivative	943
43	23.84	5514	33702117	2,4-Dimethoxyphenylpyruvic acid, di-TMS	840
44	24.277	5584	661992223	Glycine, N-benzoyl-, trimethylsilyl ester	951
45	24.38	5670	67539566	D-Fructose, 1,3,4,5,6-pentakis-O-(trimethylsilyl)-, O-methyloxime	931
46	24.458	5695	79429223	Quininic acid (5TMS)	861
47	24.589	5733	138763915	L-3-Methylhistidine, 2TMS derivative	890
48	24.755	5783	125662400	D-Psicose, pentakis(trimethylsilyl) ether, methyloxime (syn)	921
49	24.881	5819	56359320	D-Fructose, 1,3,4,5,6-pentakis-O-(trimethylsilyl)-, O-methyloxime	971
50	25.103	5884	50416689	-Glucose, 2,3,4,5,6-pentakis-O-(trimethylsilyl)-, o-methyloxyme, (1E)	810
51	25.201	5915	43864426	D-Fructose, 1,3,4,5,6-pentakis-O-(trimethylsilyl)-, O-methyloxime	922
52	25.33	5952	56380523	Gluconolactone (3R,4S,5R,6R)-, 4TMS derivative	804
53	25.597	6028	193690947	D-Mannose, 5TMS derivative	818
54	25.774	6055	1491164280	l-Glucose, 2,3,4,5,6-pentakis-O-(trimethylsilyl)-, o-methyloxyme, (1E	928
55	26.12	6184	21430575	β-D-Xylopyranose, 4TMS derivative	859
56	26.362	6238	570828144	Galactose, 2,3,4,5,6-pentakis-O-(trimethylsilyl)-, o-methyloxyme, (1Z	933
57	26.511	6304	99090584	L-Histidine, N,1-bis(trimethylsilyl)-, trimethylsilyl ester	875
58	26.758	6370	418959126	D-Mannitol, 6TMS derivative	961
59	26.935	6423	214300753	Galactaric acid, 6TMS derivative	848

Peak #	Retention Time (min)	Scan number	Peak Area	Tentative Identification	Match Score
60	27.124	6479	56605938	L-Tyrosine, 3TMS derivative	953
61	27.39	6556	51785324	Galactaric acid, 6TMS derivative	894
62	27.659	6635	35040390	3-α-Mannobiose, octakis(trimethylsilyl) ether (isomer 2)	829
63	27.73	6657	27301858	13β-Ethyl-3-oximinogon-4-en-17-one 3-TMS	846
64	27.901	6707	26082532	D-Gluconic acid, 6TMS derivative	948
65	28.402	6853	442698384	β-D-Glucopyranose, 5TMS derivative	938
66	28.823	6978	45650469	D-Gluconic acid, 6TMS derivative	858
67	28.94	7013	86669842	D-Gluconic acid, 6TMS derivative	965
68	29.268	7109	23495967	????	
69	29.577	7200	19605243	9H-Purine, 9-(trimethylsilyl)-2,6-bis[(trimethylsilyl)oxy]-	869
70	29.702	7235	20242911	-Glucuronic acid, 2,3,4,5-tetrakis-O-(trimethylsilyl)-, trimethylsilyl est	930
71	29.791	7264	17204900	Inositol, 1,2,3,4,5,6-hexakis-O-(trimethylsilyl)-, scyllo-	859
72	29.901	7295	53525187	????	
73	30.095	7351	22284608	7-Methylxanthine, 2TMS derivative	903
74	31.107	7638	154554047	2-Hydroxyhippuric acid, 2TMS derivative	949
75	31.703	7824	42919839	Myo-Inositol, 6TMS derivative	922
76	32.18	7943	405544994	Uric acid, N,O,O',O"-tetrakis(trimethylsilyl)-	864
77	33.532	8362	27566934	Glycine, N-[4-[(trimethylsilyl)oxy]benzoyl]-, trimethylsilyl ester	925
78	35.282	8875	24105189	????	
79	35.406	8913	41233803	????	
80	35.839	9040	44910783	Glycine, N-[4-[(trimethylsilyl)oxy]benzoyl]-, trimethylsilyl ester	936
81	38.901	9940	138973495	D-(-)-Lyxofuranose, tetrakis(trimethylsilyl) ether	877
82	40.455	10398	29460668	D-(-)-Ribofuranose, tetrakis(trimethylsilyl) ether (isomer 1)	849
83	41.349	10661	73636763	Ethylenediaminetetraacetic acid, tetrakis(trimethylsilyl) ester	897
84	42.557	11012	60859875	????	
85	43.22	11208	41900734	????	
86	54.25	14456	25570480	????	
87	55.488	14777	524712609	-Glucopyranosiduronic acid, 4-(acetylamino)phenyl 2,3,4-tris-O-(trimethylsi trimethylsilyl ester	874
88	56.245	15041	33467022	????	

Table S3. Tentative Peak Identifications for GC/MS of SRM 3672 Smoker's Urine

Peak #	Retention Time (min)	Scan number	Peak Area	Tentative Identification	Match Score
1	7.953	840	90868556	Glycine, 3TMS derivative	921
2	8.048	866	48824503	Butanedioic acid, 2TMS derivative	947
3	8.27	936	38749069	Glyceric acid, 3TMS derivative	956
4	8.56	1021	33206969	(R*,R*)-2,3-Dihydroxybutanoic acid, tris(trimethylsilyl)-	956
5	8.783	1082	51891440	Trimethylsilyl 2,3-bis[(trimethylsilyl)oxy]butanoate	959
6	8.977	1143	24133774	Serine, 3TMS derivative	921
7	9.604	1328	16585429	L-Threonine, 3TMS derivative	959
8	10.212	1506	19551777	????	
9	10.283	1527	23005744	Butanoic acid, 2,4-bis[(trimethylsilyl)oxy]-, trimethylsilyl este	925
10	10.83	1686	63773360	(R*,S*)-3,4-DIHYDROXYBUTANOIC ACID TRITMS	942
11	11.114	1771	23083169	????	
12	11.966	2020	47877587	Aminomalonic acid, tris(trimethylsilyl)-	941
13	12.727	2245	47033961	L-Threitol, 4TMS derivative	932
14	12.997	2324	320193317	meso-Erythritol, 4TMS derivative	960
15	13.628	2505	99413228	L-5-Oxoproline, , 2TMS derivative	953
16	13.801	2560	16805026	????	
17	13.967	2610	209664965	????	
18	14.097	2649	170202400	L-Threonic acid, tris(trimethylsilyl) ether, trimethylsilyl ester	940
19	14.606	2800	85144065	Erythronic acid, tetrakis(trimethylsilyl) deriv	937
20	15.177	2967	25004451	Pentanedioic acid, 2-[(trimethylsilyl)oxy]-, bis(trimethylsilyl) es	963
21	15.655	3106	93148201	1,2-Dithiane-4,5-diol, (Z)-, 2TMS derivative	945
22	16.493	3353	23881138	????	
23	16.9	3473	22066238	Pentanedioic acid, 2-[(trimethylsilyl)oxy]-, bis(trimethylsilyl) es	887
24	17.12	3536	18665207	????	
25	17.377	3613	44993306	4-Hydroxybenzeneacetic acid, 2TMS derivative	935
26	17.515	3653	21418462	Pentonic acid, 3-deoxy-2,4,5-tris-O-(trimethylsilyl)-, trimethylsily	781
27	17.725	3716	50344372	D-Arabinose, tetrakis(trimethylsilyl) ether, ethyloxime (isomer	908
28	17.843	3751	88572747	3,4,5-Trihydroxypentanoic acid, tetrakis(trimethylsilyl)-	921
29	17.951	3783	88210856	D-Arabinose, tetrakis(trimethylsilyl) ether, ethyloxime (isomer	897
30	18.025	3804	25403011	Furoylglycine, TMS derivative	852
31	18.103	3826	25797177	Pentonic acid, 3-deoxy-2,4,5-tris-O-(trimethylsilyl)-, trimethylsily	782
32	18.439	3909	38728523	????	
33	19.255	4165	31670078	????	

Peak #	Retention Time (min)	Scan number	Peak Area	Tentative Identification	Match Score
35	19.857	4343	123935152	D-(+)-Arabitol, 5TMS derivative	949
36	20.023	4393	84355069	L-Fucitol, 5TMS derivative	824
37	20.542	4543	15213911	????	
38	20.961	4668	67194046	1-Propene-1,2,3-tricarboxylic acid, tris(trimethylsilyl) ester, (Z	934
39	21.335	4775	54340576	Ribonic acid, 2,3,4,5-tetrakis-O-(trimethylsilyl)-, trimethylsilyl e	880
40	21.653	4869	26109192	2-Keto-l-gluconic acid, penta(O-trimethylsilyl)-	823
41	21.925	4949	71739374	????	
42	22.359	5076	20708554	????	
43	22.774	5201	17252963	????	
44	22.934	5246	34831097	D-(-)-Fructofuranose, pentakis(trimethylsilyl) ether (isomer 2	857
45	23.01	5268	25414736	????	
46	23.298	5351	607114235	Citric acid, 4TMS derivative	929
47	24.259	5631	352157419	Glycine, N-benzoyl-, trimethylsilyl ester	960
48	24.453	5695	120328651	Quininic acid (5TMS)	899
49	24.575	5730	131382002	????	
50	24.749	5782	101162405	D-Psicose, pentakis(trimethylsilyl) ether, methyloxime (syn)	912
51	24.883	5819	59808795	D-(-)-Fructose, pentakis(trimethylsilyl) ether, methyloxime (an	899
52	25.101	5884	54421559	d-Glucose, 2,3,4,5,6-pentakis-O-(trimethylsilyl)-, o-methyloxyme,	868
53	25.199	5912	44424139	D-(-)-Fructose, pentakis(trimethylsilyl) ether, methyloxime (sy	883
54	25.322	5948	95313010	????	
55	25.48	5995	48508343	d-Galactose, 2,3,4,5,6-pentakis-O-(trimethylsilyl)-, o-methyloxy (1E)-	871
56	25.587	6027	198437665	Galactopyranose, 5TMS derivative	927
57	25.756	6080	770863546	d-Glucose, 2,3,4,5,6-pentakis-O-(trimethylsilyl)-, o-methyloxyme,	912
58	26.297	6239	200937419	d-Glucose, 2,3,4,5,6-pentakis-O-(trimethylsilyl)-, o-methyloxyme,	933
59	26.475	6288	79752870	L-Histidine, N,1-bis(trimethylsilyl)-, trimethylsilyl ester	903
60	26.584	6321	18901277	L-Lysine, 4TMS derivative	859
61	26.726	6360	265046322	D-Mannitol, 6TMS derivative	946
62	26.916	6416	205474954	????	
63	27.116	6477	57656861	L-Tyrosine, 3TMS derivative	921
64	27.383	6555	38324865	Galactaric acid, 6TMS derivative	878
65	27.734	6658	62595302	????	
66	27.903	6707	22858718	D-Gluconic acid, 6TMS derivative	938
67	28.393	6849	352042836	β-D-Glucopyranose, 5TMS derivative	943

Peak #	Retention Time (min)	Scan number	Peak Area	Tentative Identification	Match Score
68	28.94	7012	113869560	D-Gluconic acid, 6TMS derivative	945
69	29.277	7113	62256369	????	
70	29.701	7236	26306220	D-Glucuronic acid, 2,3,4,5-tetrakis-O-(trimethylsilyl)-, trimethyl ester	952
71	29.902	7295	16304141	????	
72	30.088	7350	20199838	7-Methylxanthine, 2TMS derivative	906
73	31.068	7638	30415572	2-Hydroxyhippuric acid, 2TMS derivative	966
74	31.346	7721	17454001	N-Acetyl-D-glucosamine, tetrakis(trimethylsilyl) ether, methylox (syn)	845
75	31.704	7825	27080296	Myo-Inositol, 6TMS derivative	905
76	32.173	7962	382409992	Uric acid, N,O,O',O''-tetrakis(trimethylsilyl)-	851
77	33.537	8364	49596006	Trimethylsilyl ((3-[(trimethylsilyl)oxy]benzoyl)amino)acetate	915
78	35.282	8876	23739893	????	
79	35.412	8915	24150640	????	
80	35.853	9045	37033961	Glycine, N-[4-[(trimethylsilyl)oxy]benzoyl]-, trimethylsilyl est	957
81	38.891	9944	107491800	1,4-Anhydro-1-(2,4-bis[(trimethylsilyl)oxy]-5-pyrimidinyl)-2,3,5-t (trimethylsilyl)pentitol	822
82	40.455	10401	34695140	D-(-)-Ribofuranose, tetrakis(trimethylsilyl) ether (isomer 1)	859
83	41.351	10664	85193911	Ethylenediaminetetraacetic acid, tetrakis(trimethylsilyl) ester	885
84	42.55	11015	59989182	????	
85	43.205	11207	22714728	????	
86	55.386	14794	88417551	β-d-Glucopyranosiduronic acid, 4-(acetylamino)phenyl 2,3,4-tris (trimethylsilyl)-, trimethylsilyl ester	870

Table S4. Tentative Peak Identifications for GC/MS of SRM 3673 Non-Smoker's Urine

Peak #	Retention Time (min)	Scan Number	Peak Area	Tentative Identification	Match Score
1	7.952	840	38739069	Glycine, 3TMS derivative	946
2	8.038	868	10908014	Butanedioic acid, 2TMS derivative	958
3	8.275	937	59547216	Glyceric acid, 3TMS derivative	957
4	8.562	1021	27378969	(R*,R*)-2,3-Dihydroxybutanoic acid, tris(trimethylsilyl)-	957
5	8.774	1084	49360071	(R*,R*)-2,3-Dihydroxybutanoic acid, tris(trimethylsilyl)-	959
6	10.277	1526	39792967	Butanoic acid, 2,4-bis[(trimethylsilyl)oxy]-, trimethylsilyl ester	897
7	10.815	1684	28951077	Butanoic acid, 3,4-bis[(trimethylsilyl)oxy]-, trimethylsilyl ester	941
8	11.953	2018	49697227	Aminomalonic acid, tris(trimethylsilyl)-	947
9	12.713	2242	29682104	D-Threitol, 4TMS derivative	954
10	12.967	2315	135958914	meso-Erythritol, 4TMS derivative	931
11	13.253	2400	22534743	Salicylic acid, 2TMS derivative	893
12	13.595	2501	50650799	L-5-Oxoproline, , 2TMS derivative	952
13	14.075	2641	120645355	L-Threonic acid, tris(trimethylsilyl) ether, trimethylsilyl ester	932
14	14.375	2730	19596640	??	
15	14.597	2795	109235662	L-Threonic acid, tris(trimethylsilyl) ether, trimethylsilyl ester	930
16	15.208	2975	30067012	Pentanedioic acid, 2-(methoxyimino)-, bis(trimethylsilyl) ester	858
17	15.641	3103	87866929	1,2-Dithiane-4,5-diol, (Z)-, 2TMS derivative	953
18	16.479	3349	16399342	??	
19	17.117	3537	33163372	Tartaric acid, 4TMS derivative	914
20	17.715	3714	37080474	D-Arabinose, tetrakis(trimethylsilyl) ether, ethyloxime (isomer 2)	895
21	17.832	3747	61051715	3,4,5-Trihydroxypentanoic acid, tetrakis(trimethylsilyl)-	919
22	17.952	3782	78864797	D-Arabinose, tetrakis(trimethylsilyl) ether, ethyloxime (isomer 2)	865
23	18.092	3823	16396907	??	
24	18.414	3918	31802242	??	
25	19.245	4163	30676837	??	
26	19.328	4187	63381242	Levoglucosan, 3TMS derivative	909
27	19.851	4341	83337831	D-(+)-Arabitol, 5TMS derivative	952
28	20.018	4389	51998322	??	
29	20.988	4675	24008368	Acetamide, N-[4-[(trimethylsilyl)oxy]phenyl]-	912
30	21.337	4761	38771379	Ribonic acid, pentakis-TMS	895
31	21.914	4932	58863278	Ribonic acid, 2,3,4,5-tetrakis-O-(trimethylsilyl)-, trimethylsilyl ester	894
32	22.376	5084	41495715	??	
33	23	5268	25663948	D-(-)-Fructofuranose, pentakis(trimethylsilyl) ether (isomer 2)	769

Peak #	Retention Time (min)	Scan Number	Peak Area	Tentative Identification	Match Score
35	24.211	5622	536431138	Glycine, N-benzoyl-, trimethylsilyl ester	923
36	24.279	5642	51155480	??	
37	24.533	5718	214862863	L-3-Methylhistidine, 2TMS derivative	788
38	24.876	5821	242765093	D-(-)-Fructose, pentakis(trimethylsilyl) ether, methyloxime (anti)	839
39	25.742	6035	4696478735	d-Galactose, 2,3,4,5,6-pentakis-O-(trimethylsilyl)-, o-methyloxyme, (1E)-	896
40	26.398	6265	45004415	??	
41	26.553	6290	1886733813	d-Galactose, 2,3,4,5,6-pentakis-O-(trimethylsilyl)-, o-methyloxyme, (1Z)-	939
42	26.888	6409	261589473	D-Mannitol, 6TMS derivative	953
43	27.049	6457	207411055	??	
44	27.185	6494	28115442	Galactitol, 6TMS derivative	876
45	27.249	6516	27677492	L-Tyrosine, 3TMS derivative	932
46	27.465	6581	41918542	Galactaric acid, 6TMS derivative	871
47	27.743	6648	369540621	??	
48	27.862	6696	20317139	??	
49	27.956	6723	43418188	??	
50	28.188	6792	21296389	??	
51	28.482	6885	1224387919	β-D-Glucopyranose, 5TMS derivative	938
52	28.87	6992	72964634	??	
53	28.984	7025	120313758	D-Gluconic acid, 6TMS derivative	963
54	29.333	7128	59661956	??	
55	29.584	7203	20770238	??	
56	29.794	7263	18493181	Scyllo-Inositol, 6TMS derivative	893
57	29.922	7302	240647691	??	
58	30.12	7360	20062875	7-Methylxanthine, 2TMS derivative	898
59	31.119	7653	168406311	2-Hydroxyhippuric acid, 2TMS derivative	959
60	31.702	7823	89604873	Myo-Inositol, 6TMS derivative	943
61	32.162	7941	379433541	Uric acid, N,O,O',O"-tetrakis(trimethylsilyl)-	843
62	32.292	7999	23289795	D-(+)-Galactose, pentakis(trimethylsilyl) ether, ethyloxime (isomer 1)	815
63	33.525	8360	42252639	m-Trimethylsilyloxy(trimethylsilyl)hippurate	903
64	35.264	8871	16923707	??	
65	35.821	9035	16688197	Glycine, N-[4-[(trimethylsilyl)oxy]benzoyl]-, trimethylsilyl ester	953
66	38.87	9931	93164151	D-(-)-Ribofuranose, tetrakis(trimethylsilyl) ether (isomer 2)	880
67	40.43	10390	19179580	D-(-)-Ribofuranose, tetrakis(trimethylsilyl) ether (isomer 1)	853

Peak #	Retention Time (min)	Scan Number	Peak Area	Tentative Identification	Match Score
68	41.323	10644/10655	47648974	Edetic Acid, 4TMS derivative/Tp-Tolyl-β-D-glucuronide, tris(trimethylsilyl) ether, trimethylsilyl ester	911/865
69	42.522	11006	65960197	??	
70	55.394	14789	214499577	β-d-Glucopyranosiduronic acid, 4-(acetylamino)phenyl 2,3,4-tris-O-(trimethylsilyl)-, trimethylsilyl ester	888
71	56.201	15029	17134101	??	

Table S5. Tentative Peak Identifications for GC/MS of Candidate SRM 3674 Non-Smoker's Urine

Peak #	Retention Time (min)	Scan Number	Peak Area	Tentative Identification	Match Score
1	7.958	844	72457683	Glycine, 3TMS derivative	919
2	8.049	870	30937070	Butanedioic acid, 2TMS derivative	949
3	8.286	940	88001111	Glyceric acid, 3TMS derivative	963
4	8.572	1025	36493267	(R*,R*)-2,3-Dihydroxybutanoic acid, tris(trimethylsilyl)-	945
5	8.786	1087	64083340	(R*,R*)-2,3-Dihydroxybutanoic acid, tris(trimethylsilyl)-	959
6	8.985	1146	15559748	Serine, 3TMS derivative	910
7	10.219	1510	23102733	{4-Methoxy-2-[(trimethylsilyl)oxy]phenyl}{2-[(trimethylsilyl)oxy]phenyl}methanone	704
8	10.291	1530	24362591	Butanoic acid, 2,4-bis[(trimethylsilyl)oxy]-, trimethylsilyl ester	929
9	10.828	1688	47083995	(R*,S*)-3,4-DIHYDROXYBUTANOIC ACID TRITMS	931
10	11.122	1773	23930824	??	
11	11.97	2023	35507121	Aminomalonic acid, tris(trimethylsilyl)-	929
12	12.728	2246	42697039	L-Threitol, 4TMS derivative	938
13	12.983	2321	179637927	meso-Erythritol, 4TMS derivative	934
14	13.269	2405	33578018	Salicylic acid, 2TMS derivative	917
15	13.618	2508	72623758	L-5-Oxoproline, , 2TMS derivative	944
16	13.862	2580	16891928	??	
17	14.095	2648	153847356	L-Threonic acid, tris(trimethylsilyl) ether, trimethylsilyl ester	937
18	14.391	2735	25369635	??	
19	14.617	2801	144652887	L-Threonic acid, tris(trimethylsilyl) ether, trimethylsilyl ester	936
20	15.185	2970	21491576	Pentanedioic acid, 2-[(trimethylsilyl)oxy]-, bis(trimethylsilyl) ester	949
21	15.656	3107	97968841	1,2-Dithiane-4,5-diol, (Z)-, 2TMS derivative	944
22	16.497	3354	20389793	??	
23	17.131	3541	39936084	Tartaric acid, 4TMS derivative	913
24	17.383	3615	33586853	4-Hydroxybenzeneacetic acid, 2TMS derivative	915
25	17.518	3661	23014572	??	
26	17.734	3718	42192606	D-Arabinose, tetrakis(trimethylsilyl) ether, ethyloxime (isomer 2)	877
27	17.851	3752	74747275	3,4,5-Trihydroxypentanoic acid, tetrakis(trimethylsilyl)-	921
28	17.973	3789	81119126	??	
29	18.431	3934	51564707	??	
30	19.265	4167	32252574	??	
31	19.349	4192	83062427	Levoglucosan, 3TMS derivative	917

Peak #	Retention Time (min)	Scan Number	Peak Area	Tentative Identification	Match Score
33	20.037	4396	76476301	L-(-)-Fucose, tetrakis(trimethylsilyl) ether, methyloxime (syn)	836
34	20.464	4518	32357997	L-(-)-Fucose, tetrakis(trimethylsilyl) ether, methyloxime (anti)	845
35	20.98	4673	64148048	Aconitic acid, (Z)-, 3TMS derivative	919
36	21.361	4786	69420099	2-Keto-l-gluconic acid, penta(O-trimethylsilyl)-	844
37	21.934	4953	94598865	??	
38	22.418	5094	39745006	??	
39	23.026	5273	38936832	??	
40	23.327	5326	916159906	Citric acid, 4TMS derivative	928
41	24.261	5633	701634267	Hippuric acid, TMS derivative	903
42	24.343	5664	37896714	3-(3-Hydroxyphenyl)-3-hydroxypropionic acid, tris-(O-trimethylsilyl)-	784
43	24.466	5697	79845865	??	
44	24.576	5730	185640286	L-3-Methylhistidine, 2TMS derivative	859
45	24.878	5821	264489912	D-(-)-Fructose, pentakis(trimethylsilyl) ether, methyloxime (anti)	848
46	25.399	5959	133773030	??	
47	25.742	6077	4962078969	d-Glucose, 2,3,4,5,6-pentakis-O-(trimethylsilyl)-, o-methyloxyme, (1E)-	932
48	26.62	6331	2091278059	d-Glucose, 2,3,4,5,6-pentakis-O-(trimethylsilyl)-, o-methyloxyme, (1Z)-	946
49	26.963	6431	332120697	D-Mannitol, 6TMS derivative	948
50	27.116	6476	271948687	??	
51	27.239	6512	35036287	Galactitol, 6TMS derivative	882
52	27.307	6532	36063877	L-Tyrosine, 3TMS derivative	918
53	27.518	6595	65112020	Galactaric acid, 6TMS derivative	871
54	27.627	6627	19343682	Scyllo-Inositol, 6TMS derivative	827
55	27.782	6673	382331055	??	
56	27.903	6707	30517555	D-Gluconic acid, 6TMS derivative	880
57	27.992	6734	61593415	D-Gluconic acid, 6TMS derivative	922
58	28.522	6855	1485368919	D-Glucose, 5TMS derivative	900
59	28.848	6985	59534800	??	
60	28.91	7004	55257198	??	
61	29.026	7038	185922274	D-Gluconic acid, 6TMS derivative	950
62	29.347	7132	65972094	??	
63	29.625	7213	32289504	7-(Trimethylsilyl)-2,6-bis[(trimethylsilyl)oxy]-7H-purine	856

Peak #	Retention Time (min)	Scan Number	Peak Area	Tentative Identification	Match Score
64	29.814	7267	43255882	Scyllo-Inositol, 6TMS derivative	776
65	29.993	7321	735331020	??	
66	30.157	7371	28330470	7-Methylxanthine, 2TMS derivative	898
67	31.16	7371	227331367	7-Methylxanthine, 2TMS derivative	903
68	31.352	7723	17261599	N-Acetyl-D-glucosamine, tetrakis(trimethylsilyl) ether, methyloxime (syn)	837
69	31.654	7810	69050004	??	
70	31.73	7832	81588506	Myo-Inositol, 6TMS derivative	875
71	32.189	7950	429260111	Uric acid, N,O,O',O''-tetrakis(trimethylsilyl)-	845
72	32.316	8005	24824586	D-(+)-Talose, pentakis(trimethylsilyl) ether, ethyloxime (isomer 2)	797
73	33.545	8366	49031913	m-Trimethylsilyloxy(trimethylsilyl)hippurate	903
74	35.283	8878	20721163	??	
75	35.839	9040	24224356	Glycine, N-[4-[(trimethylsilyl)oxy]benzoyl]-, trimethylsilyl ester	961
76	38.887	9936	135698825	Pseudo uridine penta-tms	826
77	40.446	10395	23907632	D-(-)-Ribofuranose, tetrakis(trimethylsilyl) ether (isomer 1)	858
78	41.342	10661	62291699	??	
79	42.536	11007	68512023	??	
80	55.416	14796	256449650	??	
81	56.219	15032	24688154	4-Nitrophenyl-β-D-galacturonide, tris(trimethylsilyl) ether, trimethylsilyl ester	789
82	56.368	15076	15357418	Octaethylene glycol, 2TMS derivative	910

Table S6. Tentative Peak Identifications for GC/MS of SRM 3671, Level 1, Non-Smoker's Urine

Peak #	Retention Time (min)	Peak Area	Scan Number	Tentative Identification	Match Score
1	7.944	24289220	840	Glycine, 3TMS derivative	907
2	8.036	29631789	867	Succinic acid (2TMS)	949
3	8.276	22119084	937	Glyceric acid, 3TMS derivative	947
4	8.568	31403463	1023	(R*,R*)-2,3-Dihydroxybutanoic acid, tris(trimethylsilyl)-	958
5	8.782	49756951	1086	R*,R*)-2,3-Dihydroxybutanoic acid, tris(trimethylsilyl)-	959
6	8.988	24534126	1146	Serine, 3TMS derivative	939
7	9.616	19947364	1331	L-Threonine, 3TMS derivative	931
8	10.299	43592176	1532	Butanoic acid, 2,4-bis[(trimethylsilyl)oxy]-, trimethylsilyl ester	909
9	10.613	22926148	1625	??	
10	10.793	46399037	1678	Oxalic acid, 2TMS derivative	874
11	10.839	36369313	1691	R*,S*)-3,4-Dihydroxybutanoic acid, tris(trimethylsilyl)-	907
12	11.138	15623845	1779	??	
13	11.996	173652576	2023	Aminomalonic acid, tris(trimethylsilyl)-	951
14	12.746	54779999	2251	L-Threitol, 4TMS derivative	931
15	13.006	180214957	2321	meso-Erythritol, 4TMS derivative	949
16	13.057	23511249	2346	??	
17	13.137	39165754	2366	??	
18	13.643	182004804	2503	L-5-Oxoproline, , 2TMS derivative	956
19	14.091	799094278	2615	??	
20	14.145	185958192	2662	L-Threonic acid, tris(trimethylsilyl) ether, trimethylsilyl ester	908
21	14.669	335022598	2805	Creatinine - 3TMS Derivative	883
22	15.202	26344290	2974	??	
23	15.68	98105634	3115	1,2-Dithiane-4,5-diol, (Z)-, 2TMS derivative	944
24	16.517	37232236	3360	??	
25	16.687	27305108	3409	L-Glutamic acid, 3TMS derivative	883
26	17.159	28738368	3549	Tartaric acid, 4TMS derivative	915
27	17.403	38700341	3620	4-Hydroxybenzeneacetic acid, 2TMS derivative	948
28	17.554	20647564	3670	??	
29	17.766	25471340	3727	DL-Arabinose, tetrakis(trimethylsilyl) ether, methyloxime	904
30	17.883	112250069	3762	3,4,5-Trihydroxypentanoic acid, tetrakis(trimethylsilyl)-	937
31	17.989	52201624	3793	DL-Arabinose, tetrakis(trimethylsilyl) ether, methyloxime	919
32	18.134	18688896	3835	??	
33	18.465	27817722	3931	??	

Peak #	Retention Time (min)	Peak Area	Scan Number	Tentative Identification	Match Score
35	19.365	80334813	4196	Levoglucosan, 3TMS derivative	920
36	19.893	129856993	4352	D-(+)-Arabitol, 5TMS derivative	951
37	20.054	53023619	4401	??	
38	20.565	16146486	4549	D-(-)-Lyxofuranose, tetrakis(trimethylsilyl) ether	800
39	20.986	40787699	4674	Aconitic acid, (Z)-, 3TMS derivative	921
40	21.34	88357262	4778	2-Keto-l-gluconic acid, penta(O-trimethylsilyl)-	923
41	21.678	32509442	4878	??	
42	21.921	38611484	4945	2-Keto-l-gluconic acid, penta(O-trimethylsilyl)-	920
43	21.955	70694260	4961	??	
44	22.378	37117388	5084	??	
45	22.477	17363447	5112	??	
46	22.801	40588163	5208	L-3-Methylhistidine, 2TMS derivative	796
47	23.31	476966203	5337	Citric acid, 4TMS derivative	940
48	23.367	60358758	5374	Isocitric acid (4TMS	823
49	24.002	15755006	5560	??	
50	24.235	306929963	5611	Glycine, N-benzoyl-, trimethylsilyl ester	931
51	24.309	50766627	5651	p-Trimethylsilyloxyphenyl-(trimethylsilyloxy)trimethylsilylacrylate	850
52	24.414	109685523	5682	D-Fructose, 1,3,4,5,6-pentakis-O-(trimethylsilyl)-, O-methyloxime	930
53	24.626	201271438	5729	L-3-Methylhistidine, 2TMS derivative	902
54	24.799	221307555	5787	D-(-)-Fructose, pentakis(trimethylsilyl) ether, methyloxime	923
55	24.913	63051485	5829	D-(-)-Fructose, pentakis(trimethylsilyl) ether, methyloxime (anti)	879
56	25.046	28468092	5867	D-(-)-Fructose, pentakis(trimethylsilyl) ether, methyloxime (syn)	803
57	25.131	32725854	5892	D-Allose, pentakis(trimethylsilyl) ether, methyloxime (syn)	810
58	25.227	53368100	5920	D-(-)-Fructose, pentakis(trimethylsilyl) ether, methyloxime (syn)	867
59	25.344	33628704	5954	??	
60	25.509	71292237	6003	d-Galactose, 2,3,4,5,6-pentakis-O-(trimethylsilyl)-, o-methyloxyme, (1E)-	939
61	25.715	122284058	6064	d-Glucose, 2,3,4,5,6-pentakis-O-(trimethylsilyl)-, o-methyloxyme, (1Z)-	910
62	26.03	19025940	6157	??	
63	26.226	29784895	6213	d-Galactose, 2,3,4,5,6-pentakis-O-(trimethylsilyl)-, o-methyloxyme, (1E)-	856

Peak #	Retention Time (min)	Peak Area	Scan Number	Tentative Identification	Match Score
64	26.322	26009387	6243	D-(+)-Talose, pentakis(trimethylsilyl) ether, methyloxime (syn)	786
65	26.507	114438518	6298	L-Histidine, N,1-bis(trimethylsilyl)-, trimethylsilyl ester	901
66	26.792	466761492	6382	D-Mannitol, 6TMS derivative	945
67	26.964	210418165	6430	Galactaric acid, 6TMS derivative	840
68	27.138	76414765	6482	L-Tyrosine, 3TMS derivative	940
69	27.415	31787350	6483	Galactaric acid, 6TMS derivative	872
70	27.756	35585060	6665	13β-Ethyl-3-oximinogon-4-en-17-one 3-TMS	848
71	27.835	17311682	6688	1H-Indole-2-acetic aciid, 1-(trimethylsilyl)-, trimethylsilyl ester	840
72	27.94	44119651	6719	D-Gluconic acid, 6TMS derivative	943
73	28.831	46206266	6983	D-Gluconic acid, 6TMS derivative	896
74	28.965	61607740	7020	D-Gluconic acid, 6TMS derivative	960
75	30.119	25148372	7359	7-Methylxanthine, 2TMS derivative	929
76	31.735	55211193	7836	Myo-Inositol, 6TMS derivative	896
77	32.219	377674945	7955	Uric acid, N,O,O',O"-tetrakis(trimethylsilyl)-	848
78	33.569	35452304	8373	Glycine, N-[4-[(trimethylsilyl)oxy]benzoyl]-, trimethylsilyl ester	920
79	34.17	15961475	8550	??	
80	35.301	19897544	8881	??	
81	35.443	37191245	8945	5-Hydroxyindoleacetic acid, 3TMS derivative	930
82	35.879	26002472	9053	Glycine, N-[4-[(trimethylsilyl)oxy]benzoyl]-, trimethylsilyl ester	955
83	38.357	23396469	9781	2-O-Glycerol-α-d-galactopyranoside, hexa-TMS	971
84	38.955	166495695	9957	??	
85	39.635	17944675	10157	??	
86	40.493	27404033	100409	D-(-)-Ribofuranose, tetrakis(trimethylsilyl) ether (isomer 2)	856
87	41.401	38355750	10676	??	
88	42.541	17977869	11010	??	

Table S7. Tentative Peak Identifications for GC/MS of SRM 3671, Level 2, Non-Smoker's Urine

Peak #	Retention Time (min)	Peak Area	Scan Number	Tentative Identification	Match Score
1	7.944	270504768	838	Glycine, 3TMS derivative	921
2	8.029	88444261	865	Succinic acid (2TMS)	973
3	8.259	44024603	933	Glyceric acid, 3TMS derivative	942
4	8.77	112544456	960	??	
5	8.552	88677196	1019	(R*,R*)-2,3-Dihydroxybutanoic acid, tris(trimethylsilyl)-	949
6	8.767	111287170	1082	(R*,R*)-2,3-Dihydroxybutanoic acid, tris(trimethylsilyl)-	955
7	9.591	37534264	1325	L-Threonine, 3TMS derivative	971
8	10.216	39352432	1506	??	
9	10.27	58643132	1525	3-Deoxytetronic acid, tris-TMS	908
10	10.59	26781301	1618	??	
11	10.778	53780880	1675	Oxalic acid, 2TMS	872
12	10.815	81347518	1685	(R*,S*)-3,4-DIHYDROXYBUTANOIC ACID TRITMS	949
13	11.975	243775224	2026	Aminomalonic acid, tris(trimethylsilyl)-	949
14	12.716	105600308	2243	L-Threitol, 4TMS derivative	940
15	12.984	349835374	2322	meso-Erythritol, 4TMS derivative	950
16	13.267	55894699	2405	Salicylic acid, 2TMS derivative	916
17	13.627	120388620	2511	L-5-Oxoproline, , 2TMS derivative	957
18	14.391	28947832	2735	??	
19	14.101	316566994	2651	L-Threonic acid, tris(trimethylsilyl) ether, trimethylsilyl ester	942
20	14.603	233760976	2800	Erythronic acid, tetrakis(trimethylsilyl) deriv	911
21	15.175	59523775	2967	α-Hydroxyglutaric acid (3TMS)	951
22	15.418	20592172	3037	??	
23	15.553	25915859	3077	??	
24	15.651	107725660	3106	1,2-Dithiane-4,5-diol, (Z)-, 2TMS derivative	948
25	15.989	33127334	3206	??	
26	16.05	17891112	3225	β-Hydroxy-β-methylglutaric acid (3TMS)	874
27	16.483	48251203	3353	??	
28	16.658	80238370	3402	L-Glutamic acid, 3TMS derivative	910
29	17.122	56092047	3540	Tartaric acid, 4TMS derivative	887
30	17.377	98997422	3614	4-Hydroxybenzeneacetic acid, 2TMS derivative	957
31	17.53	127664681	3659	Gluconic acid, 2-methoxime, tetra(trimethylsilyl)-, trimethylsilyl ester	899

Peak #	Retention Time (min)	Peak Area	Scan Number	Tentative Identification	Match Score
33	17.84	205296351	3750	3,4,5-Trihydroxypentanoic acid, tetrakis(trimethylsilyl)-	917
34	17.947	135220531	3781	DL-Arabinose, tetrakis(trimethylsilyl) ether, methyloxime	897
35	18.095	29331179	3823	??	
36	18.434	134480301	3924	??	
37	19.285	47736819	4166	??	
38	19.353	237404152	4195	Levoglucosan, 3TMS derivative	884
39	19.857	258016555	4343	D-(+)-Arabitol, 5TMS derivative	955
40	20.018	144207908	4390	??	
41	20.443	27815871	4515	L-(-)-Fucose, tetrakis(trimethylsilyl) ether, methyloxime (anti)	780
42	20.54	28418168	4543	D-(-)-Lyxofuranose, tetrakis(trimethylsilyl) ether	824
43	20.955	42575685	4665	Aconitic acid, (Z)-, 3TMS derivative	910
44	21.306	123634604	4770	2-Keto-l-gluconic acid, penta(O-trimethylsilyl)-	915
45	21.91	311676144	4928	Ribonic acid, pentakis-TMS	912
46	22.345	112170855	5073	??	
47	23.302	925514696	5350	Citric acid, 4TMS derivative	935
48	24.207	344079468	5617	Glycine, N-benzoyl-, trimethylsilyl ester	928
49	24.277	88141791	5642	??	
50	24.377	198186422	5673	D-Fructose, 1,3,4,5,6-pentakis-O-(trimethylsilyl)-, O-methyloxime	882
51	24.457	129460350	5734	??	
52	24.589	177238715	5733	L-3-Methylhistidine, 2TMS derivative	885
53	24.786	386347172	5792	D-Psicose, pentakis(trimethylsilyl) ether, methyloxime (syn)	888
54	24.859	231923561	5814	??	
55	25.331	286066115	5951	Ethyl α-D-glucopyranoside, 4TMS derivative	917
56	25.927	2613237188	6078	d-Glucose, 2,3,4,5,6-pentakis-O-(trimethylsilyl)-, o-methyloxyme, (1E)-	945
57	26.404	882187964	6245	d-Glucose, 2,3,4,5,6-pentakis-O-(trimethylsilyl)-, o-methyloxyme, (1Z)-	940
58	26.748	381317345	6370	D-Mannitol, 6TMS derivative	938
59	26.968	578443595	6430	??	
60	27.05	39077924	6457	Dulcitol, 6TMS derivative	848
61	27.105	88788072	6487	??	
62	27.394	117035455	6558	??	
63	27.9	124289568	6707	D-Gluconic acid, 6TMS derivative	936

Peak #	Retention Time (min)	Peak Area	Scan Number	Tentative Identification	Match Score
64	28.383	317059158	6850	D-Glucose, 5TMS derivative	972
65	28.648	62513600	6925	D-Glucuronic acid, 2,3,4,5-tetrakis-O-(trimethylsilyl)-, trimethylsilyl ester	892
66	28.981	658924368	7013	D-Gluconic acid, 6TMS derivative	950
67	29.275	56571643	7112	??	
68	29.576	28953154	7201	Xanthine, 3TMS derivative	890
69	29.78	47119458	7260	Inositol, 1,2,3,4,5,6-hexakis-O-(trimethylsilyl)-, scyllo	864
70	29.886	36374111	7291	??	
71	31.122	265559263	7356	7-Methylxanthine, 2TMS derivative	882
72	31.691	139425110	7823	Myo-Inositol, 6TMS derivative	912
73	32.178	476422053	7943	Uric acid, N,O,O',O''-tetrakis(trimethylsilyl)-	866
74	32.292	67307710	8000	D-(+)-Mannose, pentakis(trimethylsilyl) ether, ethyloxime (isomer 2)	837
75	33.53	40088296	8363	m-Trimethylsilyloxy(trimethylsilyl)hippurate	900
76	35.84	53450153	9024	D-Mannitol, 6TMS derivative	941
77	38.896	305405985	9941	Pseudo uridine penta-tms	830
78	40.455	73000829	10400	D-(-)-Ribofuranose, tetrakis(trimethylsilyl) ether (isomer 1)	853
79	41.339	83016337	10658	??	
80	41.839	28540270	10805	β-D-Glucopyranuronic acid, 5TMS derivative	876
81	42.268	28939888	10930	??	
82	42.528	75267143	11007	??	
83	55.364	99219170	14782	β-d-Glucopyranosiduronic acid, 4-(acetylamino)phenyl 2,3,4-tris-O-(trimethylsilyl)-, trimethylsilyl ester	870

Table S8. Tentative Peak Identifications for GC/MS of SRM 3671, Level 3, Smoker's Urine

Peak #	Retention Time (min)	Peak Area	Scan number	Tentative Identification	Match Score
1	7.951	93471681	842	Glycine, 3TMS derivative	921
2	8.041	52023821	869	Succinic acid (2TMS)	949
3	8.281	22367157	939	Glyceric acid, 3TMS derivative	922
4	8.572	52504781	1018	(R*,R*)-2,3-Dihydroxybutanoic acid, tris(trimethylsilyl)-/Uracil, 2TMS derivative	957/919
5	8.789	101761543	1089	(R*,R*)-2,3-Dihydroxybutanoic acid, tris(trimethylsilyl)-	960
6	8.99	41010315	1147	Serine, 3TMS derivative	1147
7	9.616	31144125	1331	L-Threonine, 3TMS derivative	956
8	10.613	34348730	1625	??	
9	10.792	36262505	1678	Oxalic acid, 2TMS derivative	866
10	10.84	52821416	1691	Butanoic acid, 3,4-bis[(trimethylsilyl)oxy]-, trimethylsilyl ester	911
11	11.13	25372621	1776	??	
12	12	200912097	2023	Aminomalonic acid, tris(trimethylsilyl)-	958
13	12.746	60980671	2252	L-Threitol, 4TMS derivative	940
14	13.013	250987829	2230	meso-Erythritol, 4TMS derivative	954
15	13.633	94961449	2512	L-5-Oxoproline, , 2TMS derivative	953
16	14.079	643690303	2624	??	
17	14.151	287669457	2665	L-Threonic acid, tris(trimethylsilyl) ether, trimethylsilyl ester	918
18	14.559	18844400	2784	5-Hydroxymethyl-2-furoic acid, 2TMS derivative	925
19	14.679	397391855	2828	Creatinine - 3TMS Derivative	872
20	15.21	43910951	2977	Pentanedioic acid, 2-[(trimethylsilyl)oxy]-, bis(trimethylsilyl) ester	965
21	15.579	26945585	3083	??	
22	15.684	113389475	3115	1,2-Dithiane-4,5-diol, (Z)-, 2TMS derivative	943
23	16.09	22549031	3234	Pentanedioic acid, 3-methyl-3-[(trimethylsilyl)oxy]-, bis(trimethylsilyl) ester	862
24	16.524	41543411	3362	??	
25	16.698	64548325	3414	L-Glutamic acid, 3TMS derivative	910
26	16.86	18307419	3462	??	
27	16.924	27427264	3480	Ibuprofen, TMS derivative	903
28	17.162	45558132	3550	Tartaric acid, 4TMS derivative	905
29	17.413	73537543	3624	4-Hydroxybenzeneacetic acid, 2TMS derivative	959

Peak #	Retention Time (min)	Peak Area	Scan number	Tentative Identification	Match Score
30	17.56	39826122	3667	Gluconic acid, 2-methoxime, tetra(trimethylsilyl)-, trimethylsilyl ester	817
31	17.764	41360394	3727	D-Arabinose, tetrakis(trimethylsilyl) ether, ethyloxime (isomer 2)	893
32	17.886	158403088	3763	3,4,5-Trihydroxypentanoic acid, tetrakis(trimethylsilyl)-	935
33	17.992	82325388	3794	d-Ribose, 2,3,4,5-tetrakis-O-(trimethylsilyl)-, O-methyloxime	926
34	18.067	18354606	3816	Furoylglycine, TMS derivative	914
35	18.135	27051084	3836	??	
36	18.475	79848594	3937	d-Ribose, 2,3,4,5-tetrakis-O-(trimethylsilyl)-, O-methyloxime	905
37	19.284	30100282	4174	??	
38	19.379	131876994	4202	Levoglucosan, 3TMS derivative	850
39	19.405	58376496	4217	Xylitol, 5TMS derivative	927
40	19.904	188206561	4355	D-(+)-Arabitol, 5TMS derivative	953
41	20.061	99142806	4403	??	
42	20.996	85630489	4678	Aconitic acid, (E)-, 3TMS derivative	938
43	21.359	59401091	4785	2-Keto-l-gluconic acid, penta(O-trimethylsilyl)-	912
44	21.672	36314041	4874	Ribonic acid, 2,3,4,5-tetrakis-O-(trimethylsilyl)-, trimethylsilyl ester	941
45	21.963	167875465	4948	Ribonic acid, 2,3,4,5-tetrakis-O-(trimethylsilyl)-, trimethylsilyl ester	945
46	22.82	67329044	5214	9H-Purin-6-ol, 2TMS derivative	960
47	22.978	41157260	5257	D-(-)-Fructofuranose, pentakis(trimethylsilyl) ether (isomer 2)	892
48	23.044	37932234	5280	??	
49	23.349	781392757	5338	Citric acid, 4TMS derivative	929
50	24.278	461403554	5642	Hippuric acid, TMS derivative	954
51	24.422	126281605	5684	D-Fructose, 1,3,4,5,6-pentakis-O-(trimethylsilyl)-, O-methyloxime	896
52	24.496	74234975	5706	??	
53	24.625	178533301	5737	L-3-Methylhistidine, 2TMS derivative	871
54	24.8	226143390	5796	D-Psicose, pentakis(trimethylsilyl) ether, methyloxime (syn)	924
55	24.885	60056769	5821	??	
56	25.346	193276955	5957	Ethyl α-D-glucopyranoside, 4TMS derivative	927
57	25.51	135743997	6004	??	
58	25.718	77840434	6065	d-Galactose, 2,3,4,5,6-pentakis-O-(trimethylsilyl)-, o-methyloxyme, (1Z)-	878
59	26.223	41565900	6213	d-Galactose, 2,3,4,5,6-pentakis-O-(trimethylsilyl)-, o-methyloxyme, (1E)-	845

Peak #	Retention Time (min)	Peak Area	Scan number	Tentative Identification	Match Score
60	26.513	120376678	6300	L-Histidine, N,1-bis(trimethylsilyl)-, trimethylsilyl ester	900
61	26.768	243676160	6374	D-Mannitol, 6TMS derivative	954
62	26.976	372274773	6436	Galactaric acid, 6TMS derivative	835
63	27.157	84917267	6489	L-Tyrosine, 3TMS derivative	895
64	27.421	62069527	6566	Galactaric acid, 6TMS derivative	889
65	27.78	81052023	6671	??	
66	27.84	34715309	6691	3-Indoleacetic acid, 2TMS derivative	879
67	27.949	80980535	6721	D-Gluconic acid, 6TMS derivative	955
68	28.663	74537353	6931	D-(+)-Galacturonic acid, 5TMS derivative	859
69	28.847	50926506	6985	??	
70	28.973	101998406	7022	D-Gluconic acid, 6TMS derivative	967
71	29.306	45077160	7119	??	
72	29.612	21057973	7212	7-(Trimethylsilyl)-2,6-bis[(trimethylsilyl)oxy]-7H-purine	878
73	29.733	21584340	7245	D-Glucuronic acid, 2,3,4,5-tetrakis-O-(trimethylsilyl)-, trimethylsilyl ester	948
74	29.825	16631956	7274	Scyllo-Inositol, 6TMS derivative	840
75	30.129	32461967	7361	7-Methylxanthine, 2TMS derivative	880
76	31.098	39892575	7647	2-Hydroxyhippuric acid, 2TMS derivative	903
77	31.378	18475004	7330	N-Acetyl-D-glucosamine, tetrakis(trimethylsilyl) ether, methyloxime (syn	831
78	31.741	34445585	7836	Myo-Inositol, 6TMS derivative	912
79	32.221	395731504	7954	Uric acid, N,O,O',O''-tetrakis(trimethylsilyl)-	855
80	35.32	39119081	8889	??	
81	35.44	31763700	8922	L-Tryptophan, 3TMS derivative	891
82	35.51	9068432	8945	5-Hydroxyindoleacetic acid, 3TMS derivative	885
83	35.887	37451915	9055	Glycine, N-[4-[(trimethylsilyl)oxy]benzoyl]-, trimethylsilyl ester	963
84	38.075	36879140	9699	Benzoylecgonine-TMS	915
85	38.96	244122148	9959	Arabinofuranose, 1,2,3,5-tetrakis-O-(trimethylsilyl)-	909
86	40.504	53140305	10412	D-(-)-Ribofuranose, tetrakis(trimethylsilyl) ether (isomer 1)	855
87	41.406	84042963	10679	Edetic Acid, 4TMS derivative	839
88	41.912	30102007	10828	??	
89	42.338	24978891	10953	??	
90	42.597	65100604	11031	??	

Peak #	Retention Time (min)	Peak Area	Scan number	Tentative Identification	Match Score
91	43.261	36320324	11222	??	
92	55.43	59806327	14801	β-d-Glucopyranosiduronic acid, 4-(acetylamino)phenyl 2,3,4-tris-O-(trimethylsilyl)-, trimethylsilyl ester	876
